# Supplementary material for: A GBF1-Dependent Mechanism for Environmentally Responsive Regulation of ER-Golgi Transport
Source: Dev Cell. 2019 Jun 3;49(5):786–801.e6. doi: 10.1016/j.devcel.2019.04.006 (PMC6764485; doi:10.1016/j.devcel.2019.04.006)
Supplement: Document S1. Figures S1–S5 [file mmc1.pdf]

Developmental Cell, Volume *49*

Supplemental Information

A GBF1-Dependent Mechanism for Environmentally Responsive Regulation of ER-Golgi Transport

Mafalda Lopes-da-Silva, Jessica J. McCormack, Jemima J. Burden, Kimberly J. Harrison-Lavoie, Francesco Ferraro, and Daniel F. Cutler

Figure S1

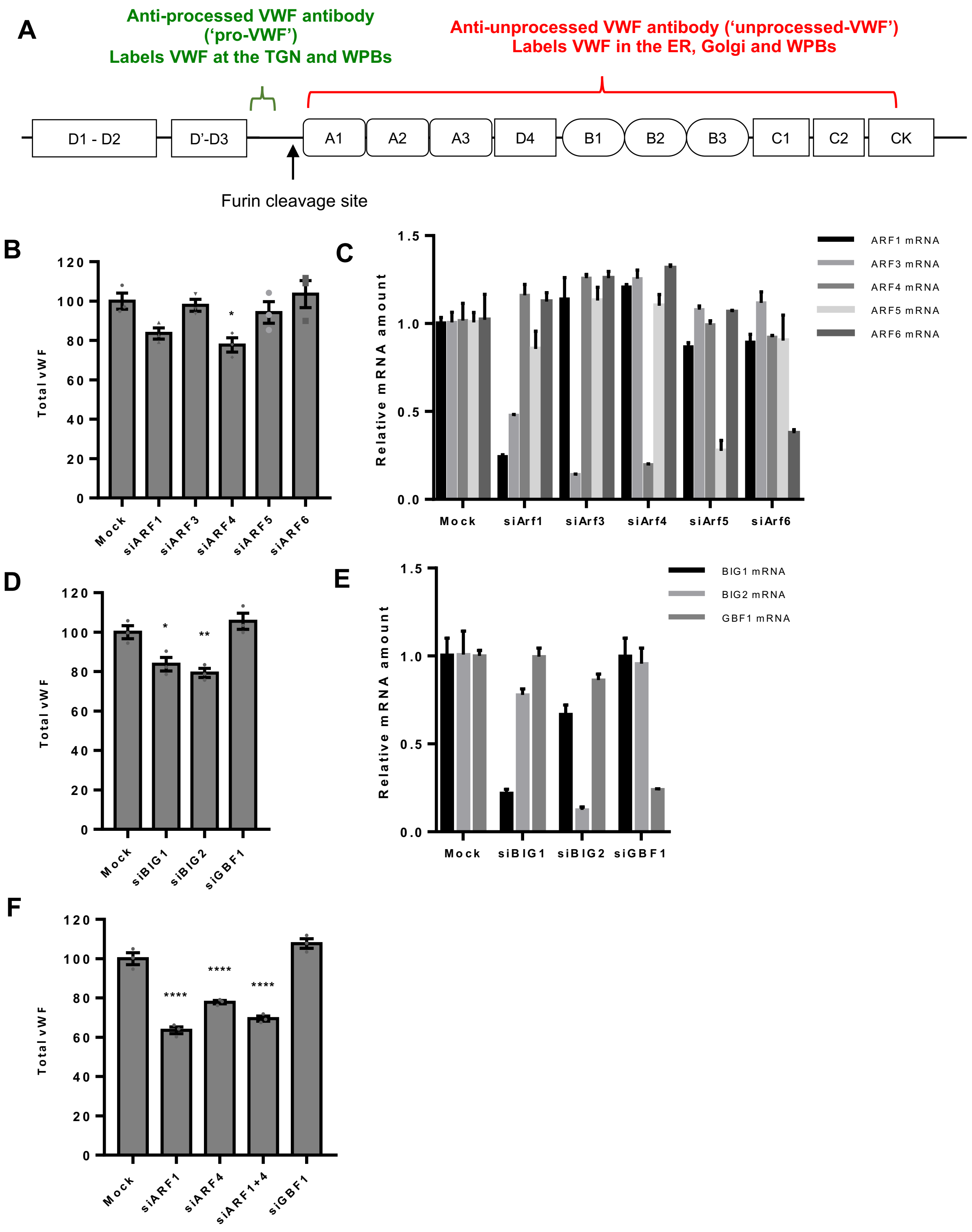
A
Anti-processed VWF antibody
('pro-VWF')
Labels VWF at the TGN and WPBs
Anti-unprocessed VWF antibody ('unprocessed-VWF')
Labels VWF in the ER, Golgi and WPBs
D1 - D2
D'-D3
A1
A2
A3
D4
B1
B2
B3
C1
C2
CK
Furin cleavage site
B
Total vWF
Mock
siARF1
siARF3
siARF4
siARF5
siARF6
C
Relative mRNA amount
ARF1 mRNA
ARF3 mRNA
ARF4 mRNA
ARF5 mRNA
ARF6 mRNA
Mock
siArf1
siArf3
siArf4
siArf5
siArf6
D
Total vWF
Mock
siBIG1
siBIG2
siGBF1
E
Relative mRNA amount
BIG1 mRNA
BIG2 mRNA
GBF1 mRNA
Mock
siBIG1
siBIG2
siGBF1
F
Total vWF
Mock
siARF1
siARF4
siARF1+4
siGBF1

Figure S1

Figure S1. ***Related to Figure 1.***

A. Diagram showing the various domains of VWF protein and antigen sites for the two different VWF antibodies used throughout this study.

B. Total amount of VWF protein in siRNA targeting ARF proteins treated cells, relative to control, measured by ELISA. N=3 independent experiments, S.E.M., one-way ANOVA with Dunnett's multiple comparisons test, *=p=0.0186.

C. rtPCR showing knockdown down of mRNA upon treatment with various siRNAs.

D. Total amount of VWF protein in siRNA targeting ARF GEF proteins treated cells, relative to control, measured by ELISA. N=3 independent experiments, S.E.M., one-way ANOVA with Dunnett's multiple comparisons test, *=p=0.0218, **=p=0.0058.

E. rtPCR showing knockdown down of mRNA upon treatment with various siRNAs.

F. Total amount of VWF protein in siRNA treated cells, relative to control, measured by ELISA. N=3 independent experiments, S.E.M., one-way ANOVA with Dunnett's multiple comparisons test, ****=P<0.0001.

Figure S2

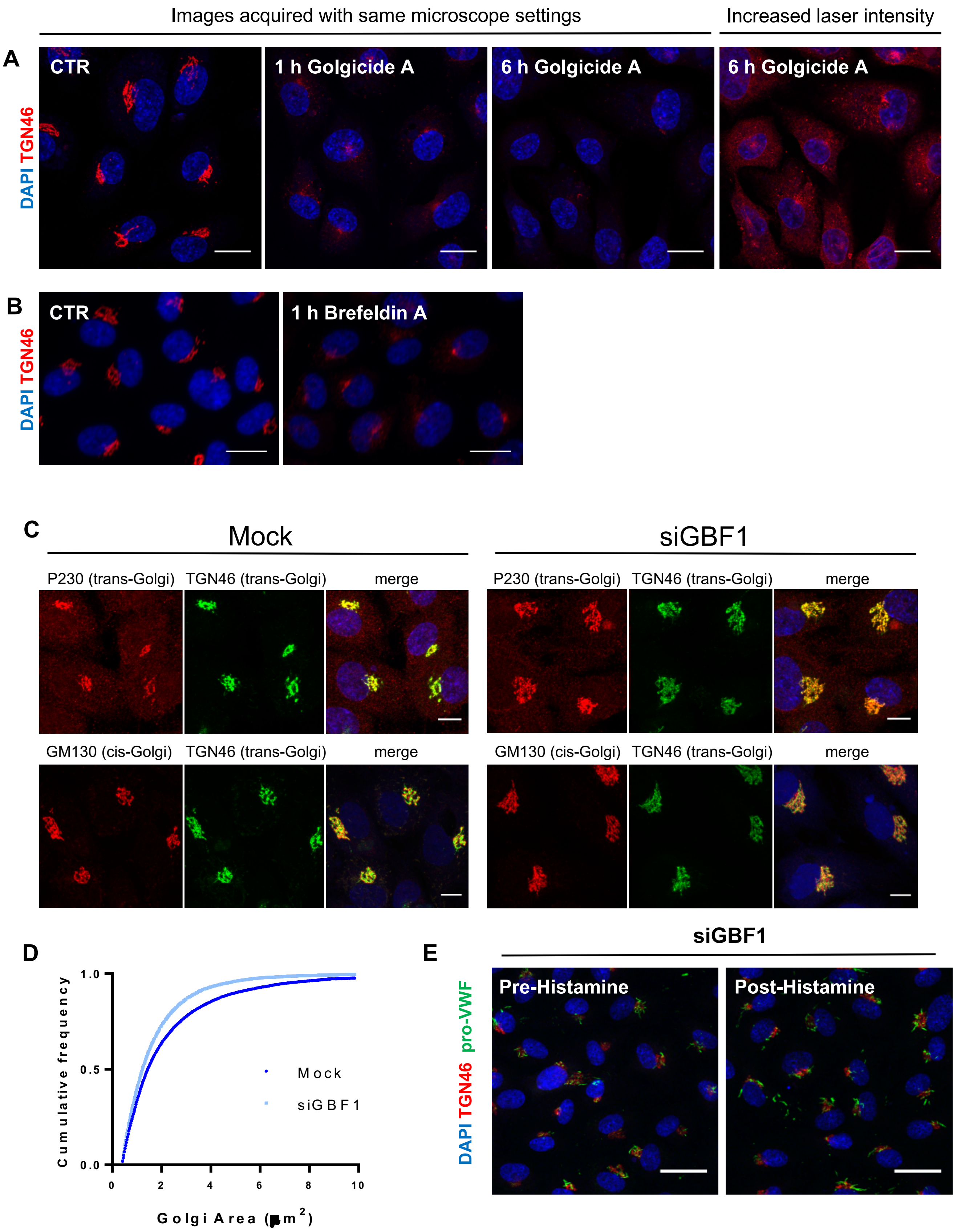

Images acquired with same microscope settings
Increased laser intensity
A
DAPI TGN46
CTR
1 h Golgicide A
6 h Golgicide A
6 h Golgicide A
B
DAPI TGN46
CTR
1 h Brefeldin A
C
Mock
siGBF1
P230 (trans-Golgi)
TGN46 (trans-Golgi)
merge
GM130 (cis-Golgi)
TGN46 (trans-Golgi)
merge
D
Cumulative frequency
1.0
0.5
0.0
0
2
4
6
8
10
Golgi Area (μm²)
Mock
siGBF1
E
siGBF1
DAPI TGN46 pro-VWF
Pre-Histamine
Post-Histamine

Figure S2

Figure S2. ***Related to Figure 1 and Figure 6.***

A. Immunofluorescence confocal images of HUVECs treated with Golgicide A for different lengths of time (hours) showing TGN46 (red) and DAPI (blue). Scale bars: 20 µm.

B. Immunofluorescence images of HUVECs treated with Brefeldin A for 1 hour showing TGN46 (red) and DAPI (blue). Scale bars: 20 µm.

C. Immunofluorescence images of control and GBF1 siRNA treated HUVECs for various ER-Golgi proteins. Scale bars: 10 µm.

D. Cumulative frequency graph showing the increase in area occupied by the Golgi in GBF1-ablated cells when compared with Mock cells. Two-sample Kolmogorov-Smirnov test, ****=P= 2.2e-16.

E. Immunofluorescence images of GBF1-ablated cells, pre and post-Histamine stimulation (30 mins), showing that most WPBs remain inside the cells, in the peri-Golgi area, and are not secreted by cells. Processed-VWF (green), TGN46 (red) and DAPI (blue). Scale bars: 30 µm.

Figure S3

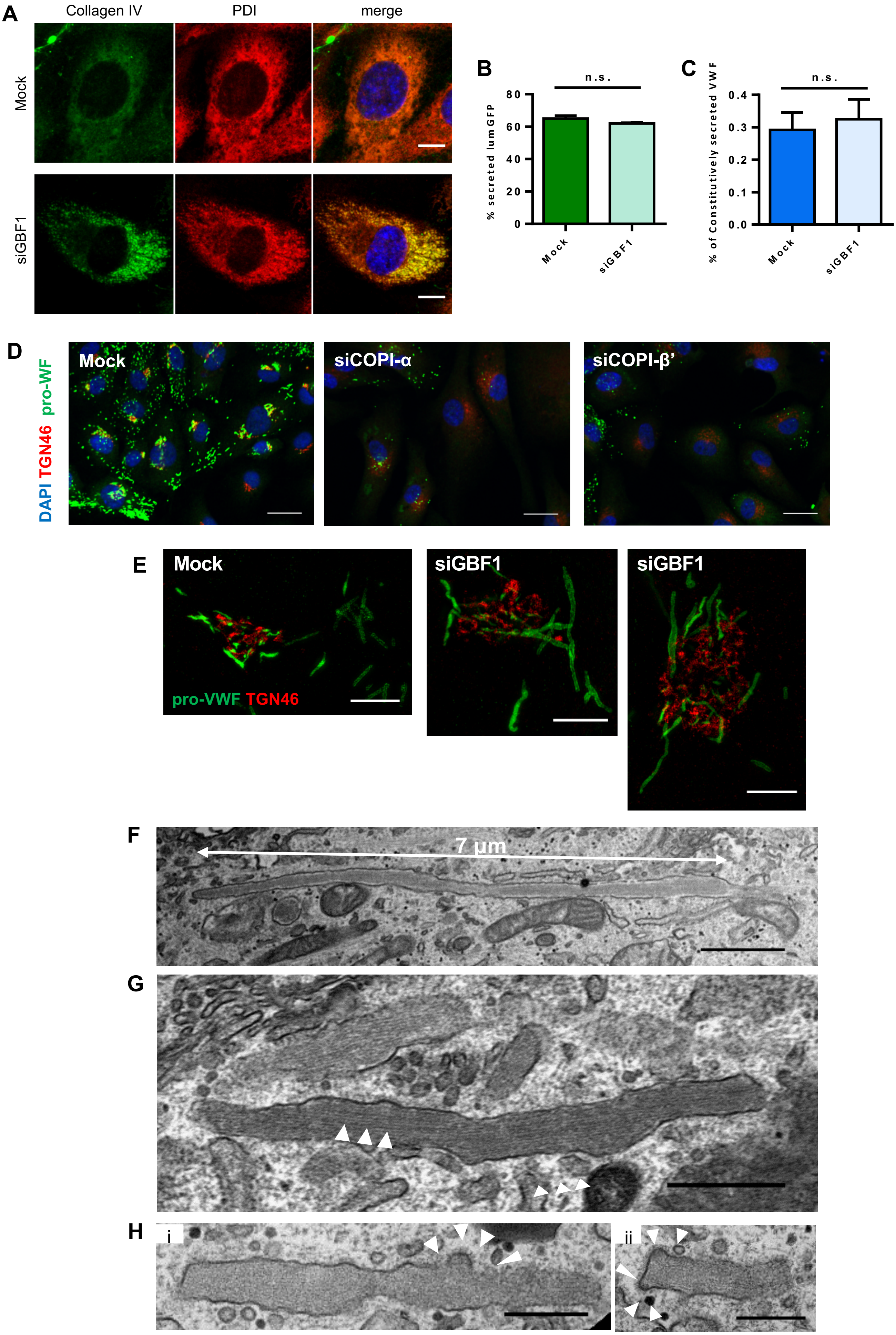
A
Collagen IV
PDI
merge
Mock
siGBF1
B
n.s.
% secreted lumGFP
80
60
40
20
0
Mock
siGBF1
C
n.s.
% of Constitutively secreted VWF
0.4
0.3
0.2
0.1
0.0
Mock
siGBF1
D
DAPI TGN46 pro-WF
Mock
siCOPI-α
siCOPI-β’
E
Mock
siGBF1
siGBF1
pro-VWF TGN46
F
7 µm
G
H
i
ii

Figure S3

Figure S3. Related to Figure 2 and Figure 5.

A. Immunofluorescence images of control and GBF1-ablated HUVECs stained with collagen IV (green) and the ER luminal marker PDI (red) and DAPI (blue). Scale bars: 20 μm.

B. Control and GBF1-ablated cells were transfected with lumGFP construct and the amount of constitutively secreted GFP was measured. N=3 independent experiments, S.E.M., T-test, n.s.= not significant.

C. Proportion of constitutively secreted VWF from total VWF in control and GBF1 siRNA treated cells. N=3 independent experiments, S.E.M., T-test, n.s.= not significant.

D. Immunofluorescence images of control, COPI-α and COPI-β’ siRNA treated HUVECs showing pro-VWF (green), TGN46 (red) and DAPI (blue). Scale bars: 30 μm.

E. Super-Resolution Structured Illumination Microscopy (SR-SIM) reconstruction of control and GBF1 siRNA treated cells stained for TGN46 (red) and processed-VWF (green) and DAPI (blue), showing cells with VWF-positive structures of varying lengths. Scale bars: 5 μm.

F. Transmission EM image of a GBF1 siRNA treated cell showing an extremely long WPB, commonly found in GBF1-ablated cells but very rarely in control cells. Scale bar: 1 μm.

G. Transmission EM image of a GBF1 siRNA treated cell showing a WPB where the VWF striations can be observed (arrows). Scale bar: 500 nm.

H. Transmission EM serial sections of a GBF1 siRNA treated cell showing area where clathrin coats are visible (arrows), suggesting that clathrin is still recruited to these WPBs. Scale bar: (i) 300 nm, (ii) 300 nm.

Figure S4

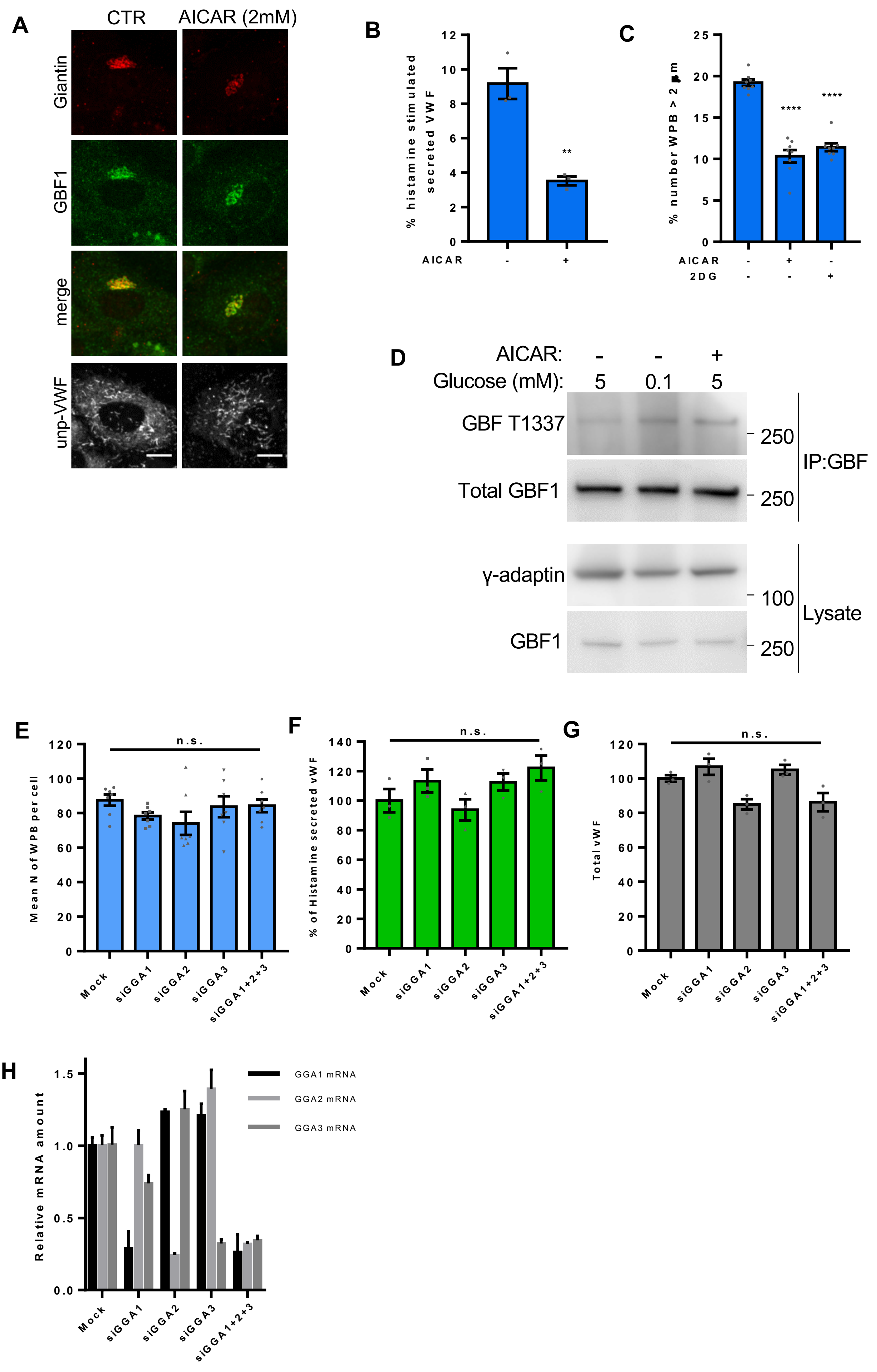
A
CTR
AICAR (2mM)
Giantin
GBF1
merge
unp-VWF
B
% histamine stimulated secreted VWF
12
10
8
6
4
2
0
**
AICAR - +
C
% number WPB > 2 µm
25
20
15
10
5
0
****
****
AICAR - + -
2DG - - +
D
AICAR: - - +
Glucose (mM): 5 0.1 5
GBF T1337
Total GBF1
250
250
IP:GBF
γ-adaptin
GBF1
100
250
Lysate
E
Mean N of WPB per cell
120
100
80
60
40
20
0
n.s.
Mock
siGGA1
siGGA2
siGGA3
siGGA1+2+3
F
% of Histamine secreted vWF
140
120
100
80
60
40
20
0
n.s.
Mock
siGGA1
siGGA2
siGGA3
siGGA1+2+3
G
Total vWF
120
100
80
60
40
20
0
n.s.
Mock
siGGA1
siGGA2
siGGA3
siGGA1+2+3
H
Relative mRNA amount
1.5
1.0
0.5
0.0
GGA1 mRNA
GGA2 mRNA
GGA3 mRNA
Mock
siGGA1
siGGA2
siGGA3
siGGA1+2+3

Figure S4

Figure S4. Related to Figure 7.

A. Immunofluorescence images of control and AICAR (2 mM) treated cells stained with giantin (red), GBF1 (green) and unprocessed-VWF (grey) antibodies. Scale bar: 0 μm.

B. HUVECS were treated with 2 mM AICAR for 24 hours prior to VWF secretion assay. Proportion of secreted VWF from total VWF in control and AICAR treated cells, upon 30 minutes histamine stimulation. N=3 independent experiments, S.E.M., unpaired T-test, **=p=0.0037.

C. HUVECs were treated with 2 mM AICAR or 5.5 mM 2-deoxuglucose (2DG) for 24 hours prior to fixation. The length of WPB was measured by HTM analysis and the graph shows the proportion of WPB in each population with a long axis longer than 2 μm. Both treatments induce the production of shorter WPBs. N=8, one-way ANOVA with Dunnett's multiple comparisons test, ****=p<0.0001.

D. Immunoprecipitation of cells treated with either control (5 mM glucose), low glucose (0.1 mM) or AICAR (2 mM).

E. Mean number of WPB per cells in siRNA targeting GGA proteins treated cells. N=7 wells where for each well the mean for each of 9 fields of view were analysed, S.E.M., one-way ANOVA with Dunnett's multiple comparisons test, n.s.= not significant.

F. Proportion of secreted VWF from total VWF in control and siRNA treated cells, upon 30 minutes histamine stimulation. Results standardised to amount secreted by control cells. N=3 independent experiments, S.E.M., one-way ANOVA with Dunnett's multiple comparisons test, n.s.= not significant.

G. Total amount of VWF protein in siRNA treated cells, relative to control, measured by ELISA. N=3 independent experiments, one-way ANOVA with Dunnett's multiple comparisons test, n.s.= not significant.

H. rtPCR showing knockdown down of mRNA upon treatment with various siRNAs.

Figure S5

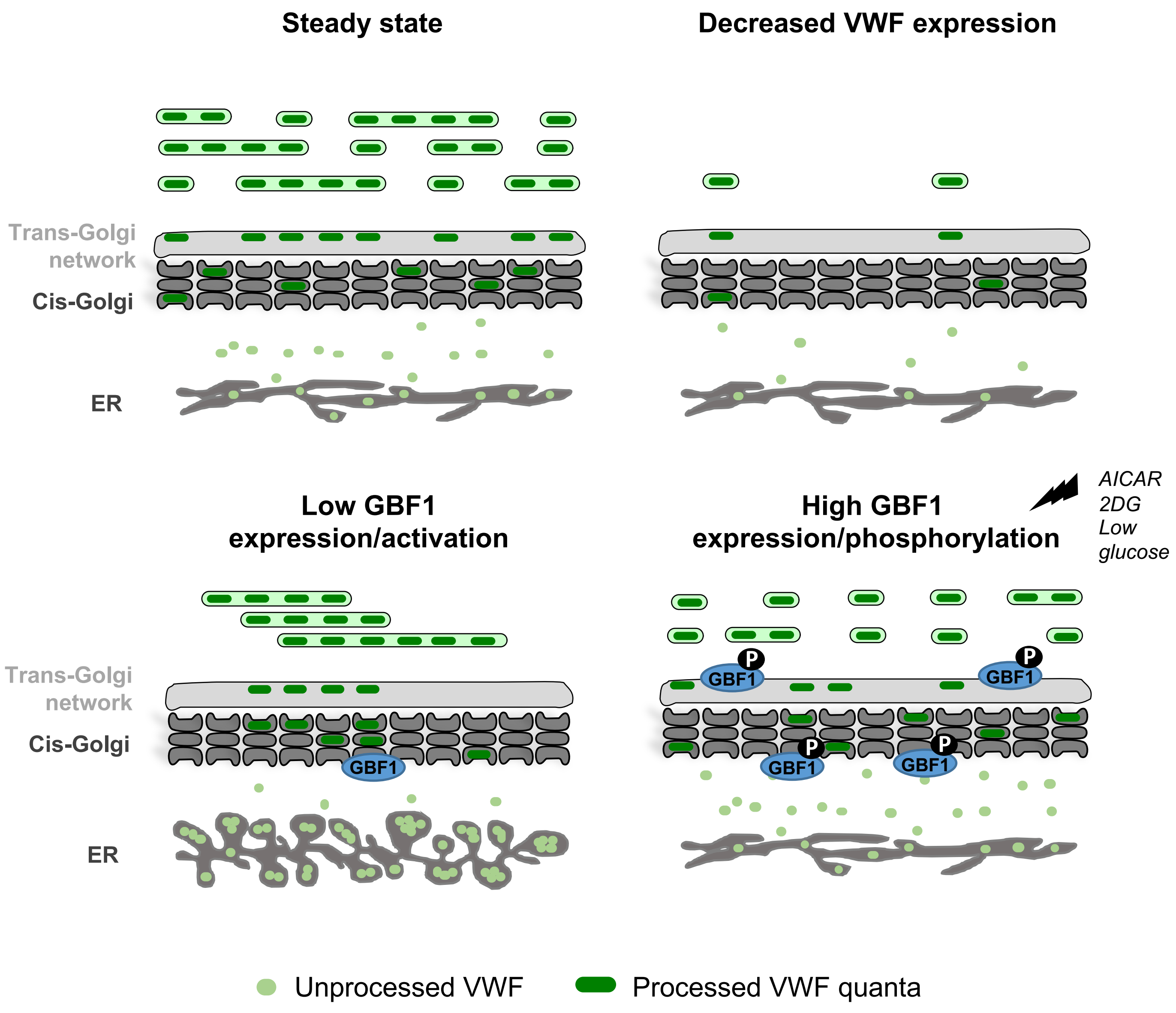

Steady state
Decreased VWF expression
Trans-Golgi network
Cis-Golgi
ER
Low GBF1 expression/activation
High GBF1 expression/phosphorylation
AICAR
2DG
Low glucose
Trans-Golgi network
Cis-Golgi
ER
GBF1
P
Unprocessed VWF
Processed VWF quanta

Figure S5

Figure S5. Related to Figures 1-7.

Model of how different sized WPBs are formed under different experimental conditions. Under steady-state conditions, VWF quanta (dark green rods) travel through the cis-Golgi (dark grey) to the continuous lumen of the TGN (light grey) and are packaged into WPBs, the size of which is determined by the number of packaged quanta. If VWF expression is lowered, then the probability of adjacent VWF quanta reaching the TGN at the same time is decreased, and more small WPBs are made. Under low GBF1 expression/activation, the number of recruited COPI-vesicles is decreased, resulting in a reduced rate of Golgi membrane retrieval and hence the rate of Golgi maturation and anterograde trafficking is also reduced. This results in a VWF exit from the ER being delayed, leading to an accumulation of unprocessed VWF (light green circles) in the ER. Since TGN progression and exit is also reduced, this leads to the accumulation of multiple VWF quanta at the TGN therefore increasing the probability of co-packaging into the same, extremely long WPB. When GBF1 is activated, via AMPK signaling (via AICAR, 2DG or glucose starvation), anterograde traffic is increased resulting in increased VWF trafficking through the ER-Golgi and the formation of smaller WPBs.
