# Supplementary material for: A GBF1-Dependent Mechanism for Environmentally Responsive Regulation of ER-Golgi Transport
Source: Dev Cell. 2019 Jun 3;49(5):786–801.e6. doi: 10.1016/j.devcel.2019.04.006 (PMC6764485; doi:10.1016/j.devcel.2019.04.006)
Supplement: Document S2. Article plus Supplemental Information [file mmc5.pdf]

Article

Developmental Cell

A GBF1-Dependent Mechanism for Environmentally Responsive Regulation of ER-Golgi Transport

Graphical Abstract

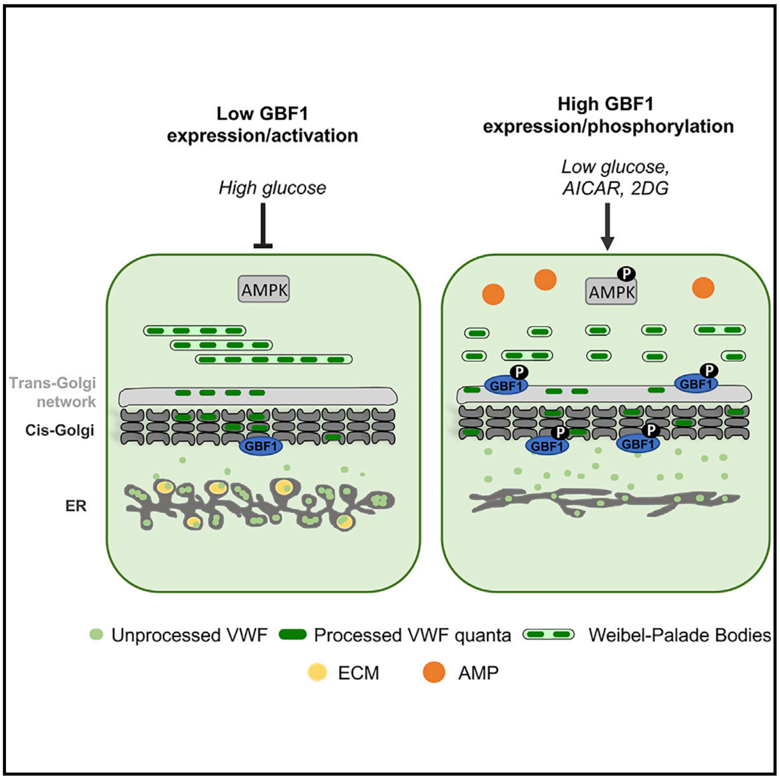

Highlights

- The Arf-GEF GBF1 modulates anterograde trafficking of VWF and ECM proteins
- Loss of GBF1 slows ER and TGN exit, producing swollen ER and giant WPBs
- Activation of GBF1 via AMPK reduces endothelial WPB size and secretion
- Metabolic change alters anterograde trafficking and cargo secretion via AMPK-GBF1

Authors

Mafalda Lopes-da-Silva,
Jessica J. McCormack,
Jemima J. Burden,
Kimberly J. Harrison-Lavoie,
Francesco Ferraro, Daniel F. Cutler

Correspondence

mafalda.silva@ucl.ac.uk (M.L.-d.-S.),
d.cutler@ucl.ac.uk (D.F.C.)

In Brief

da Silva et al. examine how the secretory pathway is coupled to environmental cues. They show that suppression of GBF1, the GEF for two Golgi Arfs (1 and 4), slows exit from the ER and the TGN. GBF1 can be phosphorylated by AMPK, and metabolic activation or inhibition of AMPK controls anterograde traffic.

Lopes-da-Silva et al., 2019, Developmental Cell *49*, 786–801
June 3, 2019 © 2019 The Authors. Published by Elsevier Inc.
https://doi.org/10.1016/j.devcel.2019.04.006

Cell Press

Developmental Cell
Article

A GBF1-Dependent Mechanism for Environmentally Responsive Regulation of ER-Golgi Transport

Mafalda Lopes-da-Silva,[1,4,*] Jessica J. McCormack,[1,3] Jemima J. Burden,[2,3] Kimberly J. Harrison-Lavoie,[1] Francesco Ferraro,[1] and Daniel F. Cutler[1,5,*]
[1]Endothelial Cell Biology Laboratory, MRC Laboratory for Molecular Cell Biology, University College London, London, UK
[2]Electron Microscopy Laboratory, MRC Laboratory for Molecular Cell Biology, University College London, London, UK
[3]These authors contributed equally
[4]Present address: CEDOC – Chronic Diseases Research Center, NOVA Medical School|Faculdade de Ciências Médicas, Universidade NOVA de Lisboa, Lisbon, Portugal
[5]Lead Contact
*Correspondence: mafalda.silva@ucl.ac.uk (M.L.-d.-S.), d.cutler@ucl.ac.uk (D.F.C.)
https://doi.org/10.1016/j.devcel.2019.04.006

SUMMARY

How can anterograde membrane trafficking be modulated by physiological cues? A screen of Golgi-associated proteins revealed that the ARF-GEF GBF1 can selectively modulate the ER-Golgi trafficking of prohaemostatic von Willebrand factor (VWF) and extracellular matrix (ECM) proteins in human endothelial cells and in mouse fibroblasts. The relationship between levels of GBF1 and the trafficking of VWF into forming secretory granules confirmed GBF1 is a limiting factor in this process. Further, GBF1 activation by AMPK couples its control of anterograde trafficking to physiological cues; levels of glucose control GBF1 activation in turn modulating VWF trafficking into secretory granules. GBF1 modulates both ER and TGN exit, the latter dramatically affecting the size of the VWF storage organelles, thereby influencing the hemostatic capacity of the endothelium. The role of AMPK as a central integrating element of cellular pathways with intra- and extra-cellular cues can now be extended to modulation of the anterograde secretory pathway.

INTRODUCTION

Effective regulation of demand-driven protein secretion requires the integration of environmental signals with cellular trafficking apparatus. On the anterograde secretory pathway, the endoplasmic reticulum (ER) and Golgi apparatus are critical cargo processing and sorting stations where multiple signals converge. Events at both must also be coordinated to effectively deliver fully-functional cargo to the correct post-Golgi destination at appropriate levels.

Human primary endothelial cells represent an effective model system for the study of anterograde trafficking control. These physiologically intact cells express a cargo—von Willebrand factor (VWF)—whose complex biosynthesis includes several measurable biosynthetic milestones. VWF undergoes dimerization in the ER prior to its (Lui-Roberts et al., 2005) transit through the Golgi to the *trans*-Golgi network (TGN) where protease cleavage, multimerization, and coiling into the proteinaceous tubules that drive the formation of its carrier Weibel-Palade body (WPB) occurs. These WPBs are endothelial-specific secretory organelles that store a variety of factors essential to primary hemostasis and inflammation. Agonist-driven exocytosis of WPBs, which causes the release of very large assemblies of VWF oligomers, is especially important in localized recruitment of platelets to sites of damage and plays a key role in primary hemostasis.

We have previously shown that WPB size depends not only on the level of VWF expression but also on the linkage of Golgi ministacks into a ribbon (Ferraro et al., 2014), which can be fragmented by statins (Ferraro et al., 2016). Importantly, the size of WPBs affects the physiological functionality of secreted VWF (Ferraro et al., 2014, 2016).

Low VWF transcription (as occurs during von Willebrand disease) (Sadler et al., 2006) leads to the production of smaller WPBs (Ferraro et al., 2014), as does overexpressing Krüppel-like Factor 2 (KLF2) (van Agtmaal et al., 2012; Ferraro et al., 2016), a transcription factor known to coordinate an anti-inflammatory and anticoagulant response (Atkins and Jain, 2007; Novodvorsky and Chico, 2014). KLF2 can be upregulated in endothelial cells by high shear stress (van Agtmaal et al., 2012; Dekker et al., 2002; Doddaballapur et al., 2015; Sathanoori et al., 2015) and tumor necrosis factor α (TNF-α) stimulation (Dekker et al., 2002). Altogether, it is clear that systems responding to cellular and extracellular cues are in place to control WPB size and ultimately regulate endothelial hemostatic and thrombotic responses.

In their active state, when bound to GTP, ADP-ribosylation factors (ARFs) play a critical role in initiating coat and downstream effector recruitment onto intracellular membranes to initiate anterograde transport vesicle formation via coat protein complex coatomer I (COPI). Mammals have 6 ARFs: ARF1–6 (humans have lost ARF2). Of relevance, ARF1 can recruit the clathrin Adaptor Protein complex-1 (AP-1) (Zhu et al., 1998, 1999) and phosphoinositide-4-kinases (PI4K), which produce phosphoinositide Phosphatidylinositol-4-phosphate (PI4P) (Godi et al., 1999), on Golgi membranes, and we have shown that both AP-1 and PI4K play an important role in WPB biogenesis (Lopes da Silva and Cutler, 2016; Lopes da Silva et al., 2016; Lui-Roberts et al., 2005). ARF activity is regulated by guanine nucleotide exchange factors (GEFs) that "switch off" ARFs by stimulating the release of bound GDP to allow for the binding of GTP and GTPase-activating

 © 2019 The Authors. Published by Elsevier Inc.
This is an open access article under the CC BY license (http://creativecommons.org/licenses/by/4.0/).

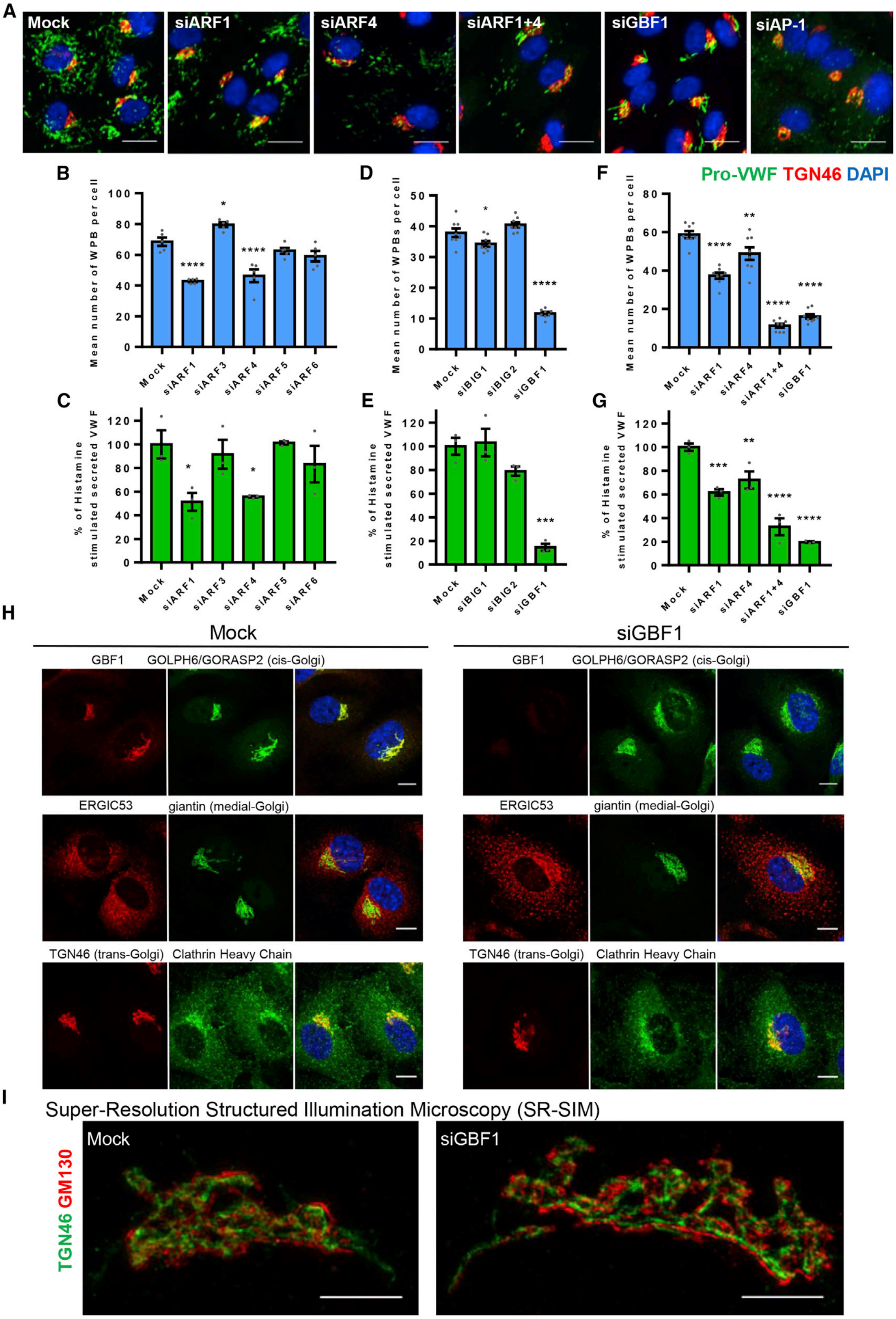

Figure 1. ARF1 and ARF4 and Their GEF GBF1 Are Involved in WPB Biogenesis

(A) Immunofluorescence images showing HUVECs treated with the indicated siRNAs. Representative images acquired using an Opera confocal and used for image quantification, stained for pro-VWF, green; TGN46, red; and DAPI, blue. Scale bars, 20 μm.

(legend continued on next page)

proteins (GAPs), which catalyze the hydrolysis of GTP to GDP, thus inactivating the ARF (Donaldson and Jackson, 2011).

We report here that the amount—as well as the phosphorylation state—of ARF GEF Golgi Brefeldin A Resistant Guanine Nucleotide Factor 1 (GBF1), acting as a GEF for ARF1 and ARF4, can control the rate of anterograde trafficking. Further, AMP-activated protein kinase (AMPK)-dependent GBF1 phosphorylation (responding to 5-Aminoimidazole-4-carboxamide ribonucleotide (AICAR) or low-glucose treatment) acts to produce smaller WPBs. Since activation of AMPK in endothelial cells can occur as a result of disturbances in blood flow (Young et al., 2009), low blood glucose (Dagher et al., 2001; Wang et al., 2012), and insulin growth factor-1 (IGF-1) stimulation (Xi et al., 2016), we conclude that GBF1 is an environmentally responsive regulator of anterograde secretory trafficking not only of VWF but also of extracellular matrix proteins (ECM). This outlines a mechanism for cells to adjust their secretory output in response to changing environmental conditions.

RESULTS

ARF1, ARF4, and Their GEF GBF1 Are Involved in WPB Biogenesis

The size of WPBs is both plastic (the length of these cigar-shaped organelles ranges from 0.5 to 5 μm) and critical to their function (Ferraro et al., 2014, 2016). We used small interfering RNA (siRNA) against human ARFs in human umbilical vein endothelial cells (HUVECs) to determine their role in WPB size control and function. We assayed for any effects on WPB biogenesis by measuring the number of WPBs per cell (using an antibody which recognizes only processed VWF inside WPBs, which we termed "pro-VWF" [Figure S1A]) using our unbiased, automated, high throughput-high content morphometric (HTM) analytical methodology (Ferraro et al., 2014), the VWF content of cells, and their exocytic response to the agonist histamine.

ARF1 is essential for AP-1 recruitment to the TGN (Zhu et al., 1998), and AP-1 is required for WPB formation (Lui-Roberts et al., 2005), but siRNA-mediated ARF1 depletion did not phenocopy AP-1 depletion since WPBs were still being formed (Figure 1A), possibly because ARF3 is known to share redundant functions with ARF1 at the late Golgi and/or TGN compartment (Manolea et al., 2010). However, ARF1 and ARF4 depletion did reduce the number of WPBs formed and their agonist-evoked exocytosis when compared to control cells (Figures 1B, 1C, S1B, and S1C). Three human ARF GEFs control ARF recruitment and activity at the ER-Golgi: BIG1, BIG2, and GBF1 (D'Souza-Schorey and Chavrier, 2006). Only ablating GBF1 similarly reduced both the number of WPBs present and their exocytic response (Figures 1A, 1D, 1E, S1D, and S1E). Since GBF1 has GEF activity for and coimmunoprecipitates with both ARF1 and ARF4 (Claude et al., 1999; Szul et al., 2007), we ablated ARF1 and ARF4 together. This gives a remarkably similar phenotype to that of GBF1-ablated cells: decreased WPB numbers per cell plus a reduced agonist response (Figures 1F, 1G, and S1F). GBF1 thus plays a major role in WPB biogenesis, likely through its combined control of ARF1 and ARF4 activation at the Golgi.

Loss of GBF1 Does Not Induce Gross Morphological Golgi Defects

GBF1 depletion could affect WPB biogenesis by changing Golgi structure. While the ARF GEF inhibitors Golgicide A or Brefeldin A (Sáenz et al., 2009) respectively cause dispersal or tubulation of the Golgi complex within 1 h (Figures S2A and S2B), GBF1-ablated HUVECs show rather a slightly extended and tubulated *cis*-Golgi as well as a more dispersed and vesicular ER-Golgi intermediate compartment (ERGIC) (Figures 1H, 1I, and S2C), overall resulting in an enlarged Golgi structure (Figure S2D). Similar effects were observed after ARF1+ARF4 double ablation (data not shown) (Volpicelli-Daley et al., 2005).

GBF1 Reduction Affects the Rate of ER Exit without Increased ER Stress

GBF1-ablated cells contained 70% fewer structures containing processed VWF (which occurs upon reaching the TGN) (Figures 1D and 1F), despite an increase in VWF mRNA (Figure 2A), but no change in VWF protein levels (Figure 2B). Where is the remaining VWF? Western blotting using an antibody recognizing all forms of VWF (termed "unprocessed-VWF") showed that (Figure 2C, VWF bands) the amount of processed VWF halved in GBF1-ablated cells (Figure 2D), indicating a fall in the transit to the TGN

(B) Mean number of WPB per cell in control and ARF siRNA treated cells. n = 5 wells, where for each well the mean for each of the 9 fields of view were analyzed, SEM., one-way ANOVA with Dunnett's multiple comparisons test, *$p < 0.5$, **$p < 0.05$, ***$p < 0.005$, ****$p < 0.0005$.

(C) Proportion of secreted VWF from total VWF in control and ARF siRNA treated cells upon 30 min histamine stimulation. Results standardized to the amount secreted by control cells in each experiment. n = 3 independent experiments, SEM., one-way ANOVA with Dunnett's multiple comparisons test, *$p < 0.5$, **$p < 0.05$, ***$p < 0.005$, ****$p < 0.0005$.

(D) Mean number of WPB per cell in control and ARF GEF siRNA treated cells. n = 5 wells, where for each well the mean for each of the 9 fields of view were analyzed, SEM., one-way ANOVA with Dunnett's multiple comparisons test, *$p < 0.5$, **$p < 0.05$, ***$p < 0.005$, ****$p < 0.0005$.

(E) Proportion of secreted VWF from total VWF in control and ARF GEF siRNA treated cells upon 30 min histamine stimulation. Results standardized to the amount secreted by control cells in each experiment. n = 3 independent experiments, SEM., one-way ANOVA with Dunnett's multiple comparisons test, *$p < 0.5$, **$p < 0.05$, ***$p < 0.005$, ****$p < 0.0005$.

(F) Mean number of WPB per cell in control and ARF1 and ARF4 and GBF1 siRNA treated cells. n = 5 wells, where for each well the mean for each of the 9 fields of view were analyzed, SEM., one-way ANOVA with Dunnett's multiple comparisons test, *$p < 0.5$, **$p < 0.05$, ***$p < 0.005$, ****$p < 0.0005$.

(G) Proportion of secreted VWF from total VWF in control and ARF1 and ARF4 and GBF1 siRNA treated cells upon 30 min histamine stimulation. Results standardized to the amount secreted by control cells in each experiment. n = 3 independent experiments, SEM., one-way ANOVA with Dunnett's multiple comparisons test, *$p < 0.5$, **$p < 0.05$, ***$p < 0.005$, ****$p < 0.0005$.

(H) GBF1 depletion in endothelial cells does not induce gross morphological Golgi defects but causes dispersion of the ERGIC. Immunofluorescence confocal images of control and GBF1-siRNA-treated HUVECs for various ER-Golgi proteins. Scale bars, 10 μm.

(I) Super-resolution structured illumination microscopy (SR-SIM) reconstruction of control and GBF1-siRNA-treated cells stained for TGN46, green and GM130, red. Scale bars, 5 μm.

See also Figures S1 and S2.

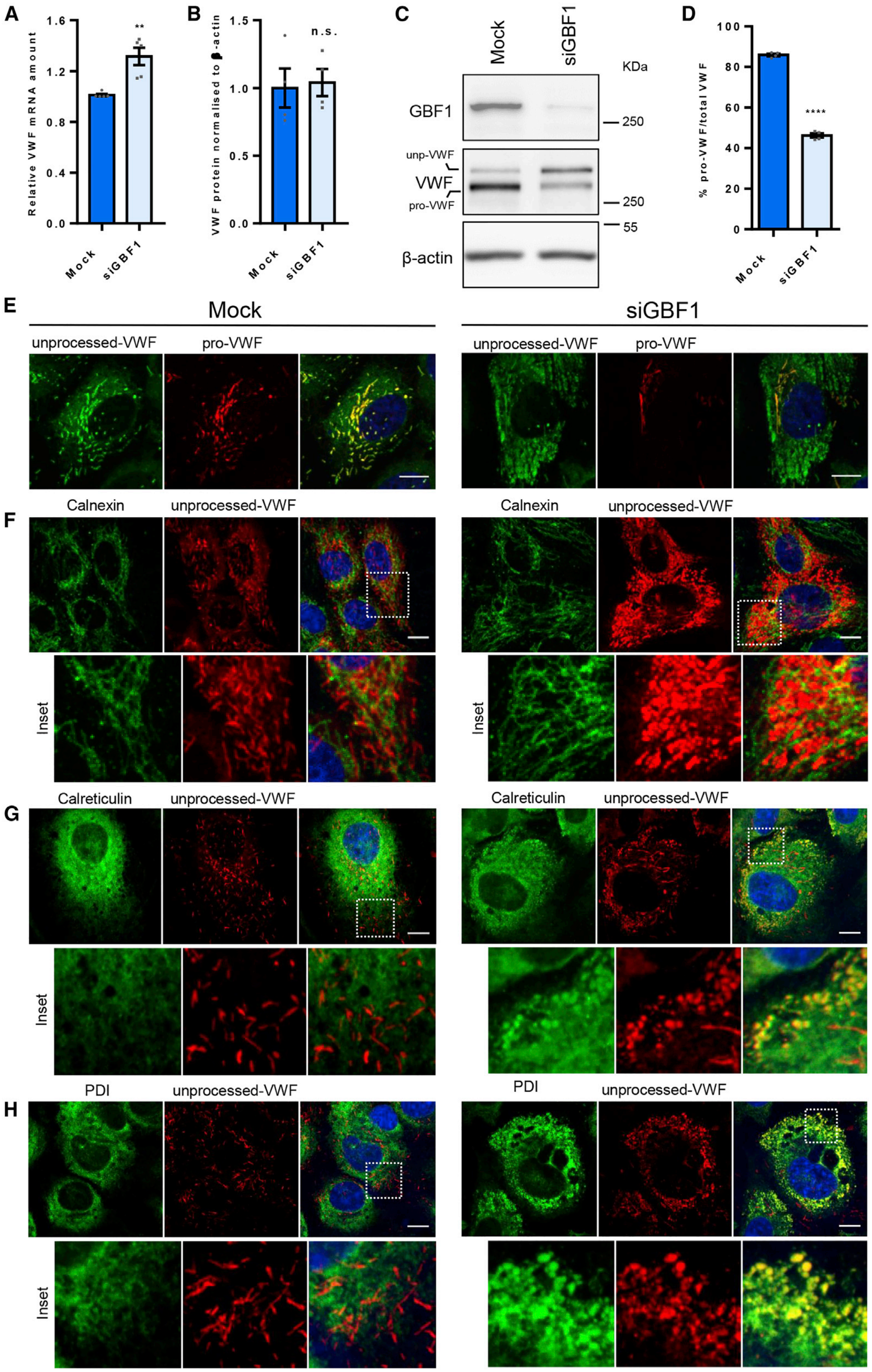

(legend on next page)

and suggesting an accumulation elsewhere. Confocal analysis of GBF1-ablated cells showed unprocessed VWF accumulating within structures distributed throughout the cytoplasm (Figure 2E). The ER transmembrane protein calnexin did not colocalize with this pool of VWF, although it does seem to surround the unprocessed VWF structures (Figure 2F). In contrast, the luminal ER markers calreticulin and oxidoreductase-protein disulfide isomerase (PDI) colocalize with these VWF structures (Figures 2G, 2H, and S3A). Correlative light and electron microscopy (CLEM) clearly show that the unprocessed-VWF accumulates in swollen interconnected, possibly specialized elements of the ER (Figure 3; Video S1), rarely observed in control cells. This suggests that GBF1 ablation reduces VWF exit from the ER.

To determine whether the GBF1-modulated rate of ER exit only affected VWF, we analyzed the distribution of another large cargo, collagen IV, which undergoes significant post-translational modifications and the secretion of which is modulated in cell division, angiogenesis, and vascular remodeling. Collagen IV colocalizes with unprocessed VWF in GBF1-ablated HUVECs (Figure 4A), and GBF1 ablation has similar effects in mouse fibroblasts (Acton et al., 2014) on collagen I, III, IV, VI, and fibronectin (Figure 4B). Thus, GBF1's role in ER-Golgi trafficking is restricted neither to VWF nor to a single cell type. Interestingly, two constitutively secreted proteins from endothelial cells, non-processed VWF (Lopes da Silva and Cutler, 2016) and lumGFP (Hannah et al., 2005), were not affected by GBF1 ablation (Figures S3B and S3C), suggesting that not all secretory cargoes are effected.

If GBF1 is acting by recruiting COPI vesicles, we hypothesized that ablation of COPI components would lead to a similar phenotype to that of GBF1 ablation. However, endothelial cells did not survive COPI ablation (Figure S3D), again suggesting that reduction of GBF1 does not cause a complete collapse and dysregulation of anterograde traffic.

Reminiscent of our current findings, targeted depletion of the machinery required for ER exit of collagen also lead to its accumulation within the ER (Nogueira et al., 2014), driving increased ER stress and upregulation of the unfolded protein response (UPR) (Wilson et al., 2011). However, RNA sequencing (RNA-seq) analysis of GBF1-depleted cells show no upregulation of UPR proteins nor of apoptotic markers (Figure 4C; Table S1), and we saw no increase in cell death in GBF1-depleted HUVECs (data not shown). Pathway enrichment analysis (see STAR Methods) showed that GBF1-deficient cells are 15.78-fold (p value 3.32E−03) enriched in retrograde vesicle-mediated transport (Golgi-to-ER) components, including COPI coat subunits. Thus, rather than a UPR response, modulating ER-Golgi trafficking by GBF1 depletion leads to compensatory upregulation of synthesis of trafficking machinery involved in the secretory pathway.

Loss of GBF1 Affects the Rate of VWF Exit from the Golgi and Component Sorting into Nascent WPBs at the TGN

Although processed VWF is found in GBF1-ablated cells (Figure 2C), a fall in processing could reflect not only ER accumulation but also reduced passage through the Golgi and incorporation into WPBs. Measuring the number of VWF "quanta" in cells (the bolus of VWF formed at the Golgi cisternae, which is then packaged into forming WPBs) showed a significant reduction in GBF1-ablated cells (Figure 5A), confirming reduced (but not absent) anterograde trafficking of VWF through the Golgi.

Whilst GBF1-ablated cells can generate WPBs these are remarkably enlarged, peri-Golgi localized (Figures 5B and S3E). Many of these VWF-positive structures, while normally shaped, were longer than 4 μm, some even reaching 7–10 μm in length (Figure S3F), sizes never seen in control cells, where modal WPB length is 1 μm (Ferraro et al., 2014). CLEM imaging revealed that these extremely long WPBs often contain multiple bends and branches, which are very rare (albeit not completely absent) in control cells (Figure 5C; Video S2). Crucially, these mega-WPBs are functionally unresponsive to the agonists histamine (Figures 6A, 6B, and S2E) and PMA (Figure 6C).

We initially hypothesized that the intra-Golgi environment could be affected by GBF1 depletion. The *trans*-Golgi environment of low pH and raised Ca^{+} levels facilitates VWF concatamerization (Huang et al., 2008) and coiling into tubules, accompanied by Furin cleavage of its propeptide (Rehemtulla and Kaufman, 1992). However, our VWF antibody is specific for the cleaved protein and binds to WPBs formed in GBF1-ablated cells, suggesting that VWF cleavage is still occurring. Furthermore, we see the function-critical tubules formed of coiled, multimerized, proteolytically processed VWF within WPBs in GBF1-ablated cells by electron microscopy (EM) (Figure S3G), suggesting that the intra-TGN environment of low pH and raised Ca^{+} is not significantly affected by the loss of GBF1.

We then hypothesized that the peri-Golgi mega-WPBs could lack the molecular machinery needed for movement and exocytic function. Indeed, while they are separate from the Golgi, and P-selectin is still recruited to the WPB membrane, tissue plasminogen activator (t-pa), Rab27a, and CD63, (recruited by different routes (Harrison-Lavoie et al., 2006)) were absent (Figure 6I). GBF1 may therefore control WPB function by modulating their ability to recruit accessory machinery at the TGN. This lack of function is not a consequence of their enlarged size *per se* since the smaller WPBs produced while the Golgi is unlinked using nocodazole treatment (Ferraro et al., 2014) of GBF1-ablated cells are also agonist unresponsive after nocodazole washout and recovery (Figures 6D–6F).

Figure 2. GBF1 Depletion Causes an Accumulation of Unprocessed VWF in Endothelial Cells

(A) Amount of VWF mRNA (relative to mock cells). n = 5 independent experiments, SEM, unpaired t test, p = 0.002.

(B) Total amount of VWF protein measured by western blot (WB) (relative to mock cells). n = 4 independent experiments, SEM, unpaired t test, n.s. = not significant.

(C) Representative western blot showing GBF1 protein depletion and VWF processing. "pro-VWF," processed VWF and "unp-VWF," unprocessed VWF.

(D) Percentage of pro-VWF from total VWF (quantification from WB). n = 4 independent experiments, SEM, unpaired t test, p < 0.0001.

(E) Immunofluorescence images of HUVECs stained with antibodies targeting unp-VWF, green and pro-VWF, red. Scale bars, 10 μm.

(F) Immunofluorescence images of HUVECs stained for the ER-transmembrane protein calnexin, green and unp-VWF, red. Scale bars, 10 μm.

(G) Immunofluorescence images of HUVECs stained for the ER-luminal protein calreticulin, green and unp-VWF, red. Scale bars, 10 μm.

(H) Immunofluorescence images of HUVECs stained for the ER-luminal protein PDI, green and unp-VWF, red. Scale bars, 10 μm.

See also Figure S3.

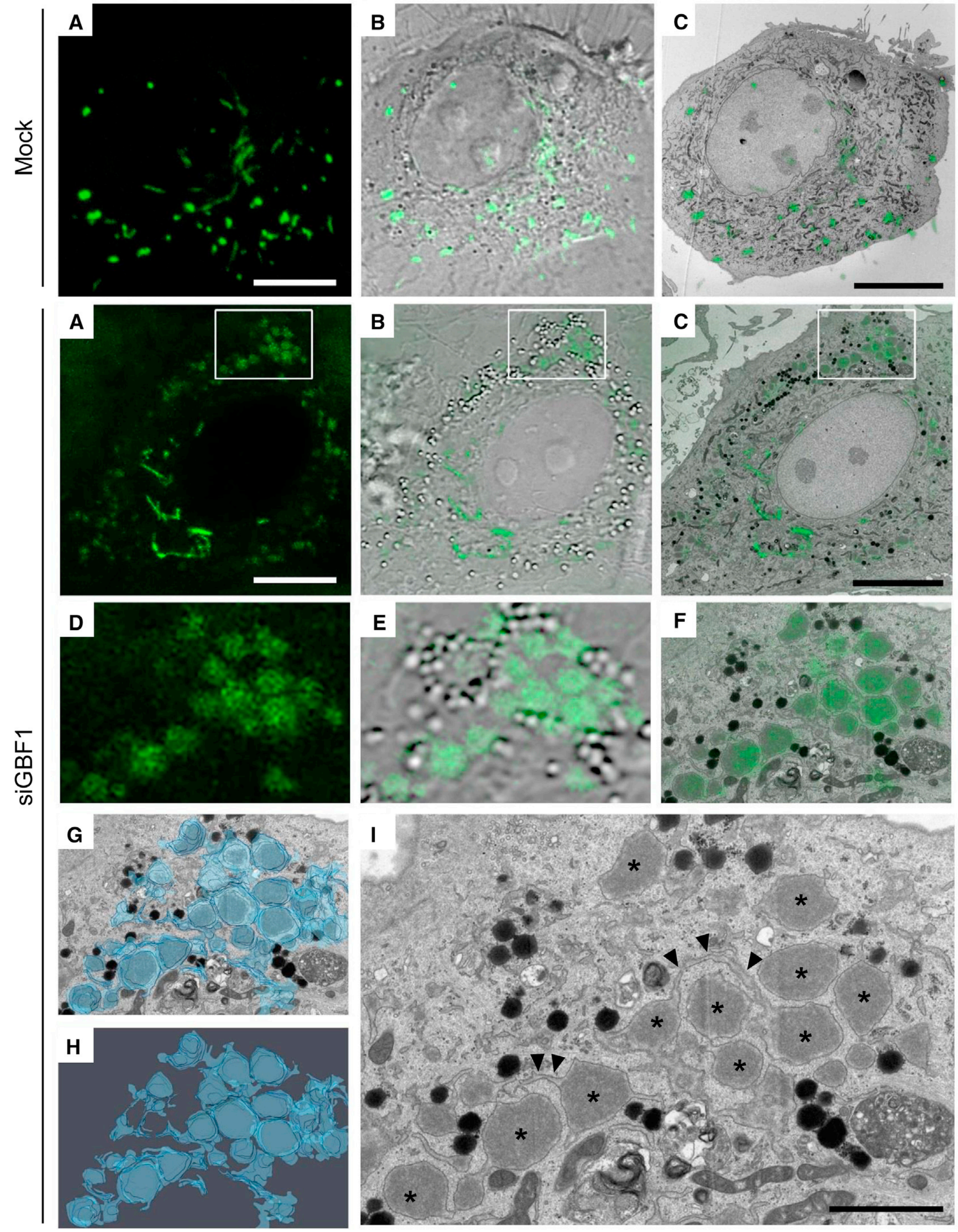

Figure 3. GBF1-Ablated Cells Accumulate Unprocessed VWF in Enlarged ER Compartments

Control and GBF1-siRNA-treated cells were processed for correlative light and electron microscopy (CLEM) analysis.

(A) Confocal image of fixed HUVECs expressing VWF-GFP. Scale bars, 10 μm.

(B) Overlay of confocal and bright field image.

(C) Overlay of confocal and single EM section. Scale bar, 10 μm.

(D) Inset of region of interest (box in i–iii), confocal image.

(E) Inset overlay of confocal and bright-field image.

(F) Inset overlay of single EM section and confocal image.

(G) Inset overlay of single EM section and 3D reconstruction from 7 EM sections.

(H) Inset 3D reconstruction from 7 EM sections.

(I) Inset single EM section showing enlarged ER compartments (*) and the various connections between them (arrowhead). Scale bar, 2 μm.

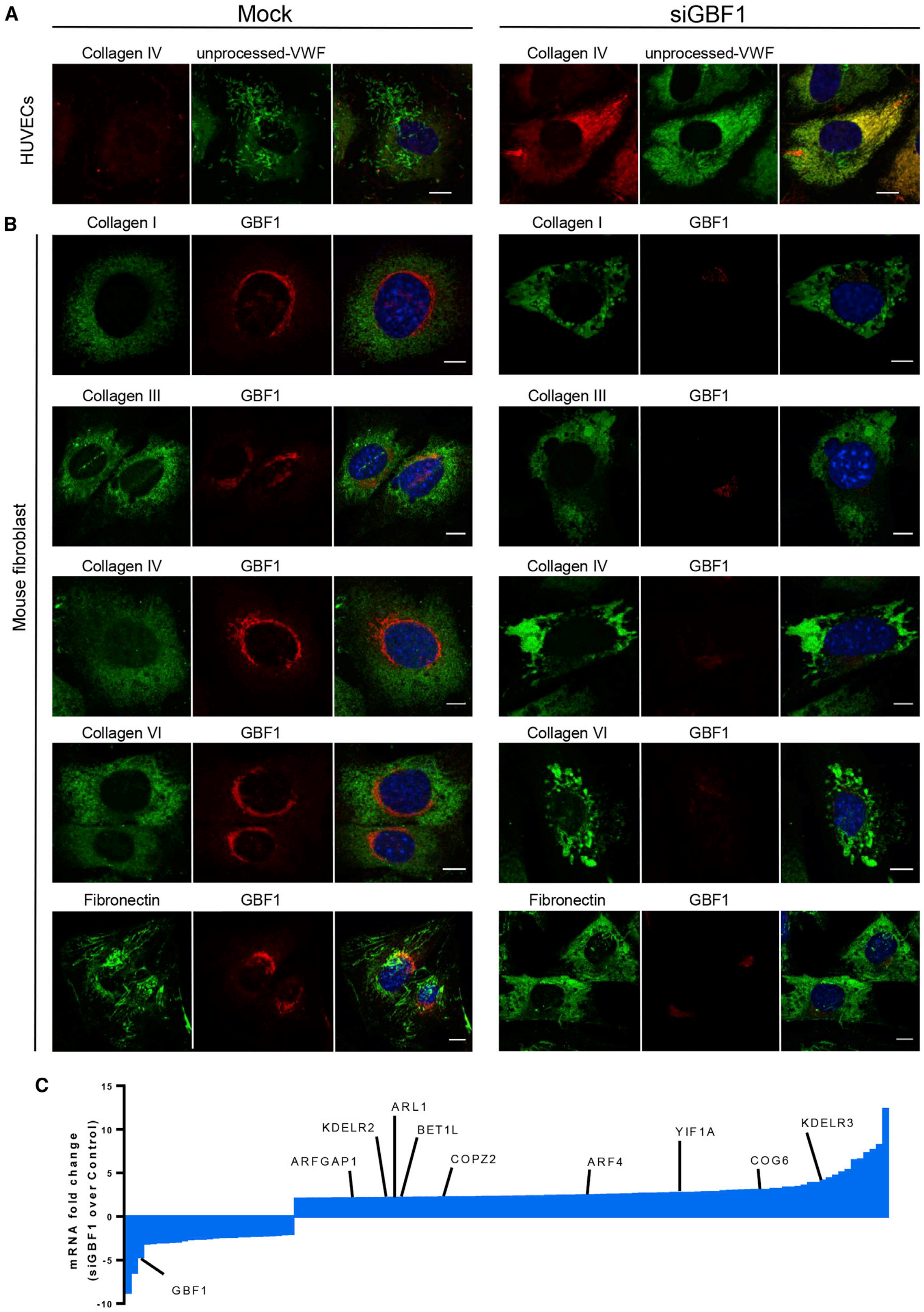

(legend on next page)

Lowering VWF protein levels by siRNA targeting of VWF generates smaller WPBs (Ferraro et al., 2014) because limiting the number of VWF ‘‘quanta’’ reaching the TGN at any given time lowers the probability of co-packaging multiple ‘‘quanta’’ into the same WPB (Figure S5). If GBF1 depletion has no effect on the rate of trafficking through the TGN, siRNAs against VWF should decrease WPB size in GBF1-deficient HUVECs to the same extent as in control cells. Instead, lowering the amount of VWF has much less effect on WPB size in GBF1-ablated cells (Figures 6G and 6H), suggesting that GBF1 affects the rate of both ER and TGN exit of VWF. Slower TGN exit should allow increased co-association of VWF ‘‘quanta’’, increasing their chance of co-packaging into forming WPB, resulting in extra-large WPBs in GBF1-depleted cells (Figure S5).

GBF1 Protein Levels and Phosphorylation Control Its Function in Golgi Trafficking

If GBF1 can affect the rate of anterograde trafficking, then do endothelial cells utilize this to control the amount and function of secretory cargo? Since GBF1 acts as a GEF for multiple ARFs, it is likely a limiting factor for downstream GEF functions. Titration of the siRNA against GBF1 (Figures 7A and 7B) revealed a dose-dependent effect on the levels of unprocessed VWF in the ER, number of VWF quanta formed by *trans*-Golgi passage, number of WPB formed, number of longer WPBs, and the level of agonist responsiveness (Figures 7C–7H). Modulating the level of GBF1 protein therefore allows endothelial cells to control the rate of VWF trafficking through the ER-Golgi and the production and size of functional WPBs.

The RNA-seq results also revealed upregulation of the transcription factor CREB3 (Table S1), which raises the transcription of anterograde trafficking components in response to cell activation (García et al., 2017). The degree of CREB3 upregulation inversely correlated with the level of GBF1 protein in cells (Figure 7I), as did other transcripts such as COPI and COPII components, consistent with GBF1 being part of a network that detects environmental changes and responds by controlling secretory capacity.

GBF1 has multiple phosphorylation sites and is a direct substrate of AMPK (Miyamoto et al., 2008), which regulates cell growth, nutrient sensing, autophagy, glucose, and lipid metabolism (Mihaylova and Shaw, 2011). Do environmental cues cause GBF1 phosphorylation to affect anterograde trafficking?

We tested this by incubating HUVECs with the AMPK activator AICAR (Miyamoto et al., 2008), which leads to phosphorylation of GBF1 in a dose-dependent manner (Figures 7J–7L). While GBF1 phosphorylation causes membrane dissociation in mitosis (Mao et al., 2013), in confluent monolayers of HUVECs, GBF1 remained on Golgi membranes even at the highest concentration of AICAR, and no Golgi fragmentation was observed (Figure S4A). AICAR causes a dose-dependent decrease in WPB size (Figure 7M) and in VWF secretion upon histamine stimulation (Figure S4B). A similar reduction in WPB size was observed by treating cells with 2 deoxy-D-glucose (2DG), another AMPK activator (Wang et al., 2011) that mimics hypoglycemia (Figure S4C).

To directly test whether AMPK affects WPB formation via GBF1, control and GBF1-ablated cells were incubated with AICAR for 24 h and the size of WPBs determined. AMPK activation results in the formation of significantly shorter WPBs in control cells, but in GBF1-ablated cells, the difference in WPB size between AICAR-treated and untreated cells was much smaller (Figures 7N and 7O), confirming that that GBF1 plays a major role in the effect of AMPK activation in the control of WPB size during its biogenesis.

Finally, we used a physiological signal to activate AMPK; treating cells with a range of low glucose concentrations (Figure S4D) (Miyamoto et al., 2008). As with AICAR, AMPK activation via decreasing glucose activated GBF1 (Figure S4D), produced shorter WPB (Figure 7P), and the size difference between control and low-glucose treatment was significantly smaller in GBF1-ablated cells, being lost in intermediate GBF1-siRNA-treated cells (Figure 7Q).

Altogether, our results suggest that extracellular signals can act through AMPK to control both the amount and activation of GBF1 to regulate ER-Golgi trafficking (Figure 7R). In endothelial cells, this has dramatic effects on the biogenesis of WPBs, modulating their size and function; this system can be used by endothelial cells to sense changing environmental conditions and modulate its hemostatic competence accordingly.

DISCUSSION

The anterograde secretory pathway is vital to homeostasis, supporting adaptation to changing environmental demands, during both developmental and (patho)physiological situations. How this coordinated response is achieved is only beginning to be unraveled (Chia et al., 2012).

Here, we show that the ARF GEF GBF1, acting via ARF1 and ARF4, modulates the rate of ER-Golgi trafficking of VWF and ECM proteins in human endothelial cells and mouse fibroblasts. GBF1 acts as a limiting factor in this process so that GBF1 translation and phosphorylation control the amount and rate of cargo secretion. While reduced GBF1 levels slow anterograde traffic, GBF1 phosphorylation speeds it up, and for WPB biogenesis, these changes have dramatic effects on organelle size and function.

GBF1 in the Early Secretory Pathway

Previous studies have shown that GBF1 controls the early secretory pathway and consistent with its localization at the *cis*-Golgi (Kawamoto et al., 2002; Zhao et al., 2002), it regulates COPI recruitment via ARF1 activation (Donaldson and Jackson, 2011; Donaldson et al., 1992; Monetta et al., 2007; Moss and Vaughan, 1995). COPI vesicles support Golgi-to-ER trafficking

Figure 4. GBF1 Depletion Causes Extra Cellular Matrix (ECM) Protein to Accumulate in the ER in HUVECs and Mouse Fibroblasts with No Activation of an ER Stress Response

(A) Control and GBF1-siRNA-treated HUVECs were stained with collagen IV, red and unprocessed-VWF, green antibodies and DAPI, blue. Scale bars, 10 μm.

(B) Control and GBF1-siRNA-treated mice fibroblasts were stained with collagen I, III, IV, VI, and fibronectin, green and GBF1, red antibodies and DAPI, blue. Scale bars, 10 μm.

(C) RNA-seq mRNA fold-change between control and GBF1-siRNA-treated HUVECs. Highlighted are proteins of interest. Full list can be found in Table S1.

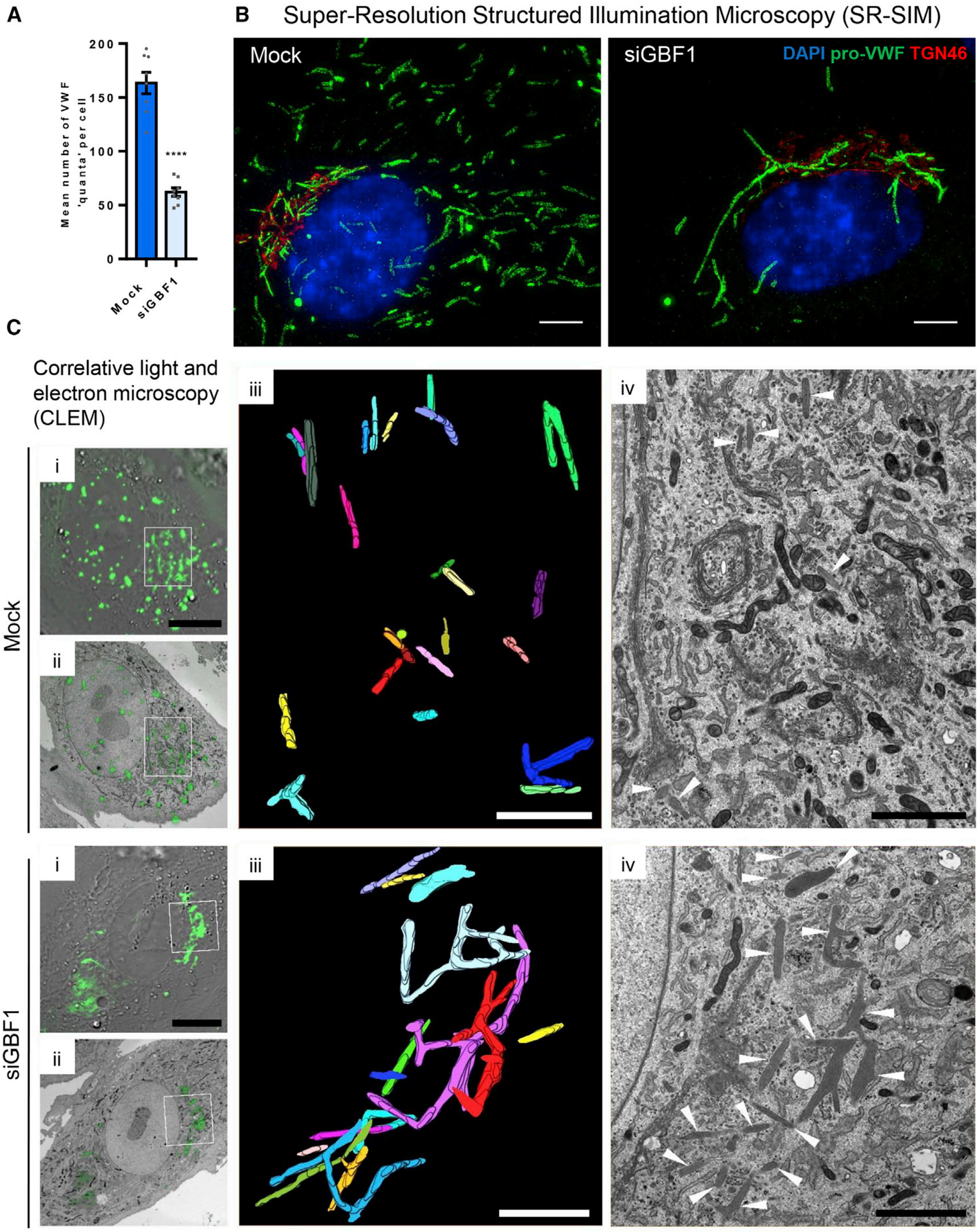

Figure 5. GBF1 depleted HUVECs produce few but very long VWF structures that remain in the peri-Golgi area

(A) Mean number of VWF "quanta" per cell in control and GBF1-siRNA-treated cells. n = 8 wells, where for each well the mean for each of the 9 fields of view were analyzed, SEM, unpaired t test, $p < 0.0001$.

(B) Super-resolution structured illumination microscopy (SR-SIM) reconstruction showing control and GBF1-siRNA-treated cells where processed-VWF, green; TGN46, red; and DAPI, blue are shown. Scale bars, 5 μm.

(C) The Golgi area, in control and GBF1-siRNA-treated cells overexpressing GFP-VWF were processed for correlative light electron microscopy (CLEM) analysis. (i) Overlay of confocal and bright field image. Scale bar, 10 μm. (ii) Overlay of confocal and a single EM section. (iii) Inset of region of interest (box in [i] and [ii]), 3D reconstruction of WPBs (mock cell 27 EM slices and siGBF1 cell, 15 EM slices). Each WPB has been pseudo-colored in a different color. Scale bar, 2 μm. (iv) Inset of region of interest, single EM section. Arrowheads point to WPBs. Scale bar, 2 μm.

See also Figure S3.

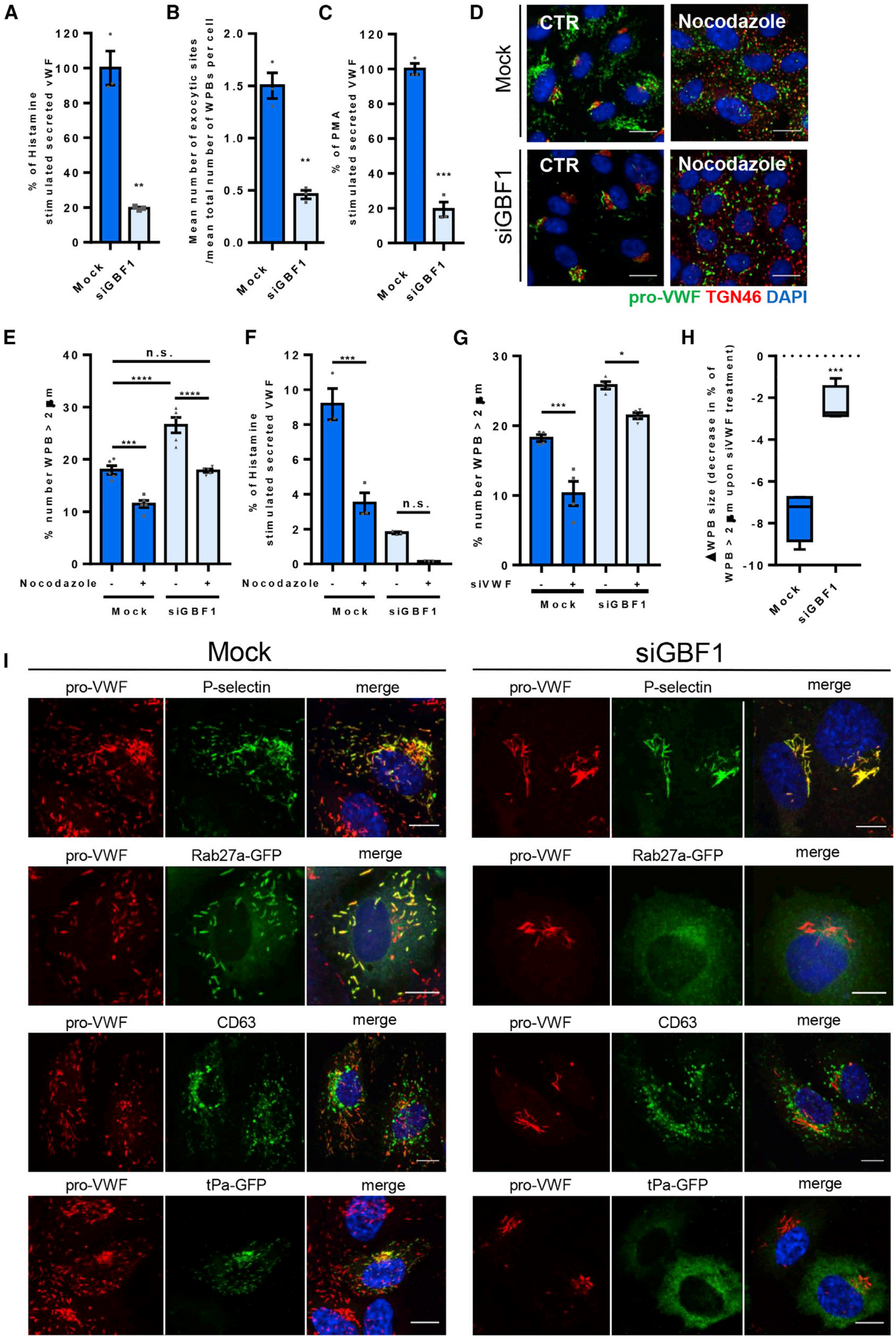

(legend on next page)

and mediate retrograde recycling of Golgi membranes, thereby controlling Golgi cisternal maturation (Ishii et al., 2016; Papanikou et al., 2015) as well as anterograde progression of cargo (Malsam et al., 2005). We now suggest that by controlling COPI recruitment via ARF1 activation, GBF1 modulates the rate of anterograde trafficking to control protein secretion and in the case of VWF and its carrier organelle (WPBs), organelle biogenesis, and VWF function (Figure S5).

While the mild alteration in *cis*-Golgi morphology and ERGIC dispersal seen in GBF1-ablated cells has been reported (Szul et al., 2007), no change in global protein secretion was seen, hinting that different cargos and cells might be differentially sensitive to GBF1 levels. Further, GBF1-depleted HeLa cells showed an increased ER retention plus an accumulation in the Golgi of vesicular stomatitis virus glycoprotein (VSV-G) (Manolea et al., 2008; Szul et al., 2007), E-selectin ligand 1 (ESL-1) (Szul and Sztul, 2011; Szul et al., 2007), and integrin-α5 (Szul and Sztul, 2011). Both VSV-G and ESL-1 eventually exit the ER and enter the Golgi in GBF1-depleted cells, but no plasma membrane arrival was observed, pointing toward a dual role for GBF1 in both ER-Golgi transport and TGN exit for some proteins (Whitt et al., 2015). A similar impairment of VSV-G trafficking was observed in cells depleted of both ARF1 and ARF4 simultaneously (Volpicelli-Daley et al., 2005), consistent with GBF1's role as a GEF for both of these proteins. More recently, GBF1 depletion in hepatocytes was found to generate extensive Golgi fragmentation (Citterio et al., 2008; Farhat et al., 2016; Hansen et al., 2017), and thus, it is not surprising that in these cells protein secretion was significantly impaired (Farhat et al., 2016) accompanied by upregulation of UPR and cell death (Citterio et al., 2008).

While the ER accumulation of both VWF and ECM proteins shown here is reminiscent of those previously seen when ER exit is blocked by depletion of components essential for this process (Boyadjiev et al., 2011; Nogueira et al., 2014; Saito et al., 2017; Santos et al., 2015; Wilson et al., 2011), this is usually accompanied by an induction of a UPR (Maiers et al., 2017; Wilson et al., 2011). Interestingly, our RNA-seq analysis showed no global translational downregulation such as is seen when the UPR is activated (only 26 transcripts were significantly downregulated in the entire transcriptome and by only 2- to 3-fold) and no UPR transcripts (e.g., Atf4, Atf5, Ddit3, Serp1, Chac1, Eif4ebp1, Hspa5, Slc7a3, atf6, xbp1, Sil1, bip, and s1p) were upregulated, even at the highest dose of GBF1 siRNA. Loss of GBF1 does not simply block ER exit to trigger a stress response but here works through a fundamentally different mechanism.

GBF1 in TGN Exit

Even at the highest dose of siRNA targeting GBF1—leaving only 10% of GBF1 protein in cells—approximately 50% of cellular VWF (Figure 2D) still exits the ER and is processed in the TGN for incorporation into (mega)-WPBs, suggesting that the Golgi/TGN lumenal environment must be largely unchanged by GBF1 depletion.

WPB formation at the TGN is AP-1 dependent; in its absence, non-multimerized, uncoiled VWF is instead secreted (Lopes da Silva and Cutler, 2016; Lui-Roberts et al., 2005) in a constitutive carrier. Despite ARF1 recruiting AP-1 to the TGN (Zhu et al., 1998), depletion of ARF1 neither alone nor in combination with ARF4 phenocopy AP-1 depletion; GBF1-depleted cells still recruit clathrin to forming WPBs (Figure S3H). GGAs also recruit clathrin to the TGN (Lefrançois and McCormick, 2007), but siRNA-mediated depletion of GGAs1-3 alone or together did not affect WPB biogenesis (Figures S4E–S4H). Sufficient clathrin must still be recruited to form WPBs by an AP-1-dependent but ARF1- and GGA-independent mechanism in GBF1-ablated cells.

How are the oversized WPBs in GBF1-depleted cells generated? Our current model of WPB biogenesis suggests that WPB length reflects numbers of VWF "quanta" (approx 570 nm in length) linearly co-packaged into each WPB at the TGN. This depends on two factors: the level of VWF expression that controls the numbers of "quanta" being made at any time, (i.e., their concentration) and, thus, the chance of quantal co-packaging and the extent of Golgi ribbon linkage since after

Figure 6. WPB Produced in GBF1-Depleted Cells Have Impaired Secretory Capacity and Lack Key Molecular Components Essential for Function

(A) Proportion of secreted VWF from total VWF, upon 30 min of histamine stimulation. Relative to mock values. n = 3 independent experiments, SEM, unpaired t test, p = 0.0012.

(B) Mean number of VWF exit sites per mean total number of WPBs per cell, upon 15 min of histamine stimulation. n = 6 wells where for each well the mean for each of 9 fields of view were analyzed, SEM, unpaired t test, $p < 0.001$.

(C) Proportion of secreted VWF from total VWF, upon 30 min PMA stimulation. Relative to mock values. n = 3 independent experiments, SEM, unpaired t test, p = 0.0001.

(D) Control and GBF1-siRNA-treated cells were treated with 1 μg/mL Nocodazole for 24 h prior to fixation. Representative images acquired using Opera confocal, showing pro-VWF, green; TGN46, red; and nucleus, blue. Scale bar, 10 μm.

(E) The length of WPB was measured by HTM imaging and the graph shows the proportion of WPB in each population with a long axis longer than 2 μm. n = 5 wells, where for each well the mean for each of the 9 fields of view were analyzed, SEM, two-way ANOVA with a Sidak's multiple comparisons test, **$p < 0.05$, ****$p < 0.0005$.

(F) Control and GBF1-siRNA-treated cells were treated with Nocodazole for 24 h prior to VWF secretion stimulation with histamine for 30 min (without the presence of nocodazole). Proportion of secreted VWF from total VWF is shown. n = 3 independent experiments, SEM, two-way ANOVA with a Sidak's multiple comparisons test, ***p = 0.0002, n.s.= not significant.

(G) Control and GBF1-siRNA-treated cells were treated with siRNA targeting VWF. The length of WPB was measured by HTM imaging and the graph shows the proportion of WPB in each population with a long axis longer than 2 μm. n = 4 wells, where for each well the mean for each of the 9 fields of view were analyzed, SEM, two-way ANOVA with a Sidak's multiple comparisons test, **$p < 0.05$, n.s. = not significant.

(H) The change in WPB size after lowering the levels of VWF by siRNA treatment, in control and GBF1-siRNA-treated cells. n = 4 independent experiments, graphs show median (with minimum and maximum), unpaired t test, p = 0.0003.

(I) Immunofluorescence images of control and GBF1-siRNA treated cells showing protein markers typically associated with functioning WPBs. Scale bars, 10 μm. See also Figure S2.

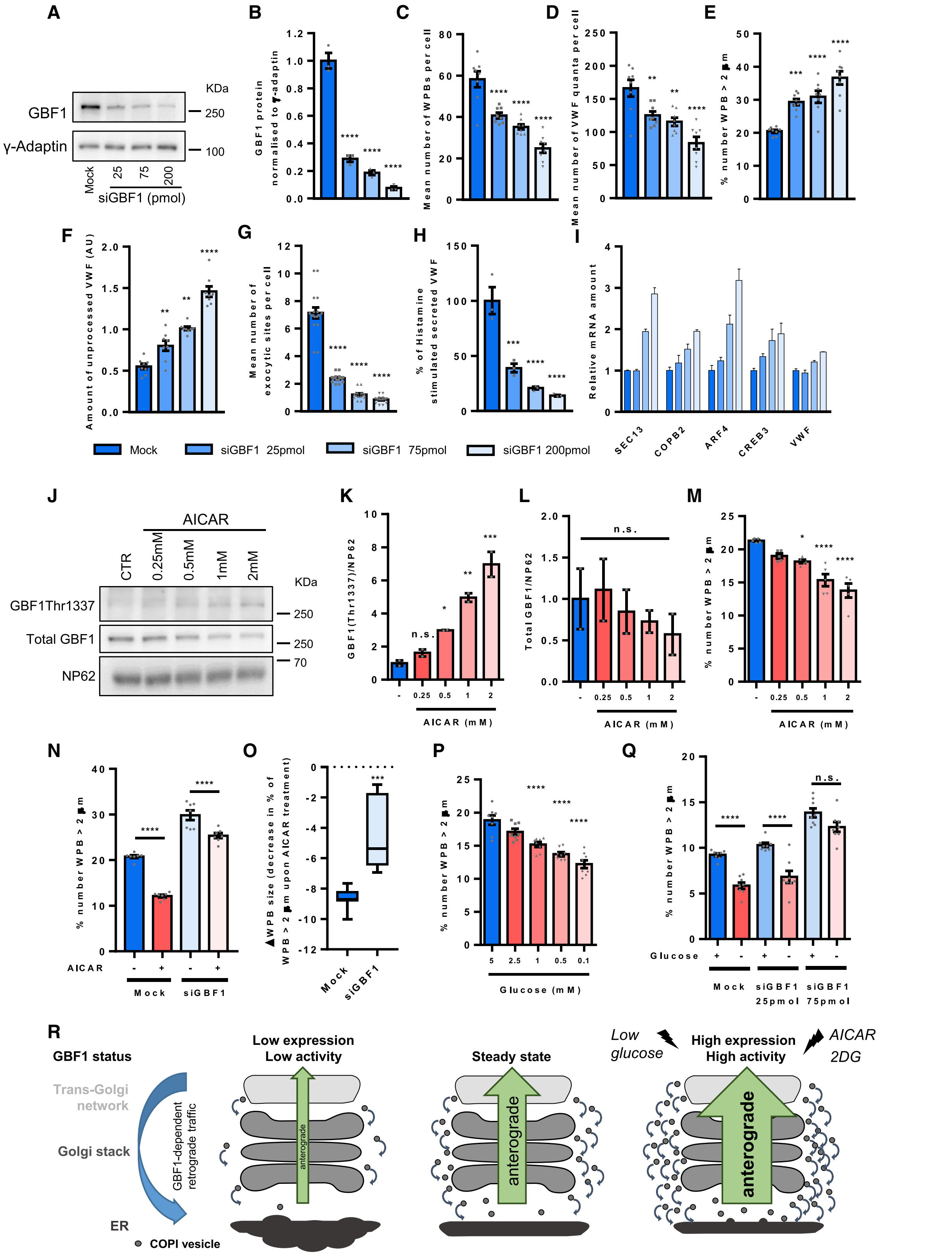

(legend on next page)

Golgi disassembly only short WPBs can form (Figure S5) (Ferraro et al., 2014). According to our model, GBF1 depletion increases VWF mRNA levels but has no effect on VWF protein levels yet reduces the amount of VWF reaching the TGN and packaged into WPBs; we would therefore expect only short WPBs to be formed. Instead, we see giant WPBs.

We speculate that giant WPBs formed in GBF1-depleted cells may reflect the reduced COPI recruitment previously reported (Malsam et al., 2005; Szul and Sztul, 2011; Szul et al., 2007), leading to a reduction in anterograde trafficking. At the *trans*-Golgi, this, exacerbated by the highly complex TGN-based processing of VWF from dimers into tubules up to 5 μm long, thus allowing for their increased co-packaging. Consistent with this, large branched WPBs arise after temporarily blocking VWF exit from the TGN by prolonged incubation at 40.5°C (data not shown). The findings of others that most secretory proteins are not similarly affected (Szul and Sztul, 2011; Szul et al., 2007) might well reflect the simpler processing requirements of most cargo.

These oversized WPBs do not respond to agonist nor do they move away from the TGN, and they lack some cargos and essential components, such as Rab27a. It has been suggested that there is an optimal *trans*-Golgi and immature granule transit time for different secretory granule cargo, crucial to correct sorting of multiple proteins into the same forming organelle (Kuliawat and Arvan, 1992). If so, then differentially slowing VWF's transit time through the TGN and into WPBs might cause proteins that transit more rapidly to fail to be recruited. Further, the reduction in COPI retrieval might also allow proteins or lipids to accumulate on the forming membranes of nascent giant WPBs, altering their final apparent identity, again potentially leading to a failure in component recruitment and secretory function. The external identity of the newly formed WPB is likely critical to recruitment of Rab27a since this depends on a content-driven, maturation-dependent mechanism that is independent of cell type (Hannah et al., 2003).

GBF1 Levels and Activity Control Its Function in Response to the External Environment

GBF1 contains between 28 and 40 phosphorylation sites (phosphosite.org). It is phosphorylated during mitosis, at Thr[1337], either by CDKI (Morohashi et al., 2010) or by AMPK (Mao et al., 2013), to dissociate it from Golgi membranes. This may partially explain the Golgi disassembly and inhibition of protein secretion seen during mitosis. During interphase,

Figure 7. GBF1 Protein Levels and Phosphorylation Control its Function in VWF Golgi Trafficking

(A) Representative blot showing the amount of GBF1 protein in cells treated with varying levels of siRNA targeting GBF1 (applies to experiments shown in (B)–(I)).

(B) Quantification of the amount of GBF1 protein in cells treated with varying levels of siRNA targeting GBF1 (quantification of WB). n = 3 independent experiments, one-way ANOVA with Dunnett's multiple comparisons test, ****$p < 0.0001$.

(C) Mean number of WPB per cell. n = 8 wells, where for each well the mean for each of the 9 fields of view were analyzed, SEM, one-way ANOVA with Dunnett's multiple comparisons test, ****$p < 0.0001$.

(D) Mean number of VWF quanta per cell. n = 8 wells, where for each well the mean for each of the 9 fields of view were analyzed, SEM, one-way ANOVA with Dunnett's multiple comparisons test, **$p < 0.01$, ****$p < 0.0001$.

(E) Proportion of WPBs longer than 2 μm in the entire WPB population. n = 8 wells, where for each well the mean for each of the 9 fields of view were analyzed, SEM, one-way ANOVA with Dunnett's multiple comparisons test, ***$p < 0.001$, ****$p < 0.0001$.

(F) Amount of unprocessed VWF, quantified by image analysis (see STAR Methods). n = 8 wells, where for each well the mean for each of the 9 fields of view were analyzed, SEM, one-way ANOVA with Dunnett's multiple comparisons test, **$p < 0.01$, ****$p < 0.0001$.

(G) Mean number of VWF exocytic sites per cell upon 15 min of histamine stimulation. n = 8 wells, where for each well the mean for each of the 9 fields of view were analyzed, SEM, one-way ANOVA with Dunnett's multiple comparisons test, ****$p < 0.0001$.

(H) Proportion of secreted VWF from total VWF upon 30 min of histamine stimulation, relative to mock cells. n = 3 independent experiments, SEM, one-way ANOVA with Dunnett's multiple comparisons test, ***$p < 0.001$, ****$p < 0.0001$.

(I) rtPCR showing changes in mRNA levels for the indicated transcript relative to mock samples.

(J) Representative blot showing the varying amounts of GBF1Thr[1337] phosphorylation in HUVECs treated with varying concentrations of AICAR for 24 h.

(K) Quantification of the amount of GBF1Thr[1337] relative to loading control (quantification of WB). n = 2 independent experiments, SD, one-way ANOVA with Dunnett's multiple comparisons test, *$p = 0.038$, **$p = 0.0021$, ***$p = 0.0003$, n.s. = not significant.

(L) Quantification of the total amount of GBF1 relative to loading control (quantification of WB). n = 2 independent experiments, SD, one-way ANOVA with Dunnett's multiple comparisons test, n.s. = not significant.

(M) Proportion of WPBs longer than 2 μm in the entire WPB population after 24 h AICAR treatment. n = 5 wells, where for each well the mean for each of the 9 fields of view were analyzed, SEM, one-way ANOVA with Dunnett's multiple comparisons test, *$p = 0.0110$, ****$p < 0.0001$.

(N) Control and GBF1-siRNA-treated cells were treated with 2 mM AICAR for 24 h. Proportion of WPBs longer than 2 μm in the entire WPB population. n = 7 wells, where for each well the mean for each of the 9 fields of view were analyzed, SEM, two-way ANOVA with a Sidak's multiple comparisons test, ****$p = 0.0001$, ***$p = 0.001$.

(O) The change in WPB size after AICAR treatment in control and GBF1-siRNA-treated cells. n = 7 independent experiments, median (with minimum and maximum), unpaired t test, *** $p = 0.0006$.

(P) Proportion of WPBs longer than 2 μm in the entire WPB population after 24 h of varying glucose treatment. n = 8 wells, where for each well the mean for each of the 9 fields of view were analyzed, SEM, one-way ANOVA with Dunnett's multiple comparisons test, ****$p < 0.0001$.

(Q) Control and GBF1-siRNA-treated cells were treated with 5 nM ("+") or 0.1 mM glucose ("−") for 24 h. Proportion of WPBs longer than 2 μm in the entire WPB population. n = 8 wells, where for each well the mean for each of the 9 fields of view were analyzed, SEM, two-way ANOVA with a Sidak's multiple comparisons test, ****$p = 0.0001$, n.s. = not significant.

(R) Model of the effect of GBF1 depletion in retrograde membrane retrieval and anterograde cargo trafficking. The levels of GBF1-dependent COPI-mediated Golgi membrane retrieval control the rates of anterograde cargo traffic through the ER-Golgi. Low levels of GBF1 in cells, reduce the amount of COPI vesicles, reducing the rate of Golgi membrane retrieval and hence the rate of Golgi maturation and anterograde trafficking. Phosphorylation of GBF1 via AICAR, 2DG, or glucose starvation increase GBF1 activity resulting in an increase in anterograde trafficking.

See also Figure S4.

phosphorylated GBF1 (at Thr^{1337}) remains associated with Golgi membranes (Miyamoto et al., 2008), suggesting that GBF1 phosphorylation has different functional, cell-cycle-dependent consequences and that AMPK-induced effects during interphase may involve GBF1's catalytic activity. Morohashi et al. (2010) also show that ARF1, which is responsible for COPI recruitment, is unaffected by GBF1 during mitosis; differential GBF1 phosphorylation may be critical to regulating different specific subcellular functions.

Many extracellular conditions modulate AMPK activation in endothelial cells (Hardie, 2004). AMPK-dependent phosphorylation of GBF1 could represent an effective mechanism to fine tune the rate of trafficking through the ER-Golgi and, in the case of endothelial cells, WPB biogenesis and function. Our data pointing toward GBF1 phosphorylation increasing anterograde trafficking are consistent with the proposal that GBF1 phosphorylation leads to prolonged ARF1 activation (Hansen et al., 2017), which could increase COPI recruitment and increase the rate of Golgi trafficking.

Recently, studies have found new splice variants (Claude et al., 2003) and cellular functions for GBF1 such as at the plasma membrane, in mitochondria, and in regulation of lipid droplets (Ackema et al., 2014; Beller et al., 2008; Busby et al., 2017; Farhat et al., 2016; Guo et al., 2008; Gupta et al., 2009; Mazaki et al., 2012; Soni et al., 2009). There is also a growing literature on GBF1 as a host factor required for virus replication (Farhat et al., 2016, 2018; Hansen et al., 2017; Lanke et al., 2009). GBF1 is probably essential in mammals and at least in zebrafish embryos, GBF1 loss of function causes disruption of vascular integrity and hemorrhage because of endothelial apoptosis (Chen et al., 2017), complicating the study of this protein in higher organisms. Because of the complexity of this large protein, the various trafficking steps it can modulate, plus the high probability that it may have cell- and temporally specific functions, it is not surprising that new and important roles for GBF1 are still being discovered. By controlling the anterograde pathway, GBF1 is essential to complex organismal survival, and here, we present a dataset outlining how this might be achieved.

STAR★METHODS

Detailed methods are provided in the online version of this paper and include the following:

- KEY RESOURCES TABLE
- CONTACT FOR REAGENT AND RESOURCE SHARING
- EXPERIMENTAL MODEL AND SUBJECT DETAILS
  - Cells
- METHOD DETAILS
  - Drugs and Drug Treatments
  - Microscopy – Antibodies Used
  - Microscopy - Confocal
  - Microscopy – Super-resolution Structured Illumination Microscopy (SR-SIM)
  - Microscopy - High-Throughput Morphometry (HTM) Analysis
  - Microscopy – Correlative Light and Electron Microscopy (CLEM)
  - siRNA Treatment
  - Quantitative PCR
  - VWF Secretion Assay
  - lumGFP Secretion and GFP ELISA
  - Western Blotting / Immunoprecipitation
  - RNAseq
  - Pathway Enrichment Analysis
- QUANTIFICATION AND STATISTICAL ANALYSIS

SUPPLEMENTAL INFORMATION

Supplemental Information can be found online at https://doi.org/10.1016/j.devcel.2019.04.006.

ACKNOWLEDGMENTS

We would like to thank Chris Stefan, Graham Warren, Steve Moss, and Tom Nightingale for insightful advice and critical reading of the manuscript. We thank Frances Brodsky for the clathrin antibody. This work was funded by the MRC (MC_UU_12018/2).

AUTHOR CONTRIBUTIONS

M.L.S. designed the study, performed the majority of the experiments, analyzed the data, and wrote the manuscript. J.M., K.H., and F.F. performed the experiments and analyzed the data. J.J.B. co-performed all the electron microscopy experiments. D.F.C. supervised the project, designed the study, interpreted the data, and wrote the manuscript. All authors contributed to the manuscript.

DECLARATION OF INTERESTS

The authors declare no competing interests.

Received: February 19, 2018
Revised: February 19, 2019
Accepted: April 4, 2019
Published: May 2, 2019

REFERENCES

Ackema, K.B., Hench, J., Böckler, S., Wang, S.C., Sauder, U., Mergentaler, H., Westermann, B., Bard, F., Frank, S., and Spang, A. (2014). The small GTPase Arf1 modulates mitochondrial morphology and function. EMBO J. *33*, 2659–2675.

Acton, S.E., Farrugia, A.J., Astarita, J.L., Mourão-Sá, D., Jenkins, R.P., Nye, E., Hooper, S., van Blijswijk, J., Rogers, N.C., Snelgrove, K.J., et al. (2014). Dendritic cells control fibroblastic reticular network tension and lymph node expansion. Nature *514*, 498–502.

van Agtmaal, E.L., Bierings, R., Dragt, B.S., Leyen, T.A., Fernandez-Borja, M., Horrevoets, A.J.G., and Voorberg, J. (2012). The shear stress-induced transcription factor KLF2 affects dynamics and angiopoietin-2 content of Weibel-Palade bodies. PLoS One *7*, e38399.

Atkins, G.B., and Jain, M.K. (2007). Role of Krüppel-like transcription factors in endothelial biology. Circ. Res. *100*, 1686–1695.

Beller, M., Sztalryd, C., Southall, N., Bell, M., Jäckle, H., Auld, D.S., and Oliver, B. (2008). COPI complex is a regulator of lipid homeostasis. PLoS Biol. *6*, e292.

Blum, R., Stephens, D.J., and Schulz, I. (2000). Lumenal targeted GFP, used as a marker of soluble cargo, visualises rapid ERGIC to Golgi traffic by a tubulo-vesicular network. J. Cell Sci. *113*, 3151–3159.

Boyadjiev, S.A., Kim, S.D., Hata, A., Haldeman-Englert, C., Zackai, E.H., Naydenov, C., Hamamoto, S., Schekman, R.W., and Kim, J. (2011). Cranio-lenticulo-sutural dysplasia associated with defects in collagen secretion. Clin Genet. *80*, 169–176.

Busby, T., Meissner, J.M., Styers, M.L., Bhatt, J., Kaushik, A., Hjelmeland, A.B., and Sztul, E. (2017). The Arf activator GBF1 localizes to plasma membrane sites involved in cell adhesion and motility. Cell. Logist. *7*, e1308900.

Cardona, A., Saalfeld, S., Schindelin, J., Arganda-Carreras, I., Preibisch, S., Longair, M., Tomancak, P., Hartenstein, V., and Douglas, R.J. (2012). TrakEM2 software for neural circuit reconstruction. PLoS One *7*, e38011.

Chen, J., Wu, X., Yao, L., Yan, L., Zhang, L., Qiu, J., Liu, X., Jia, S., and Meng, A. (2017). Impairment of cargo transportation caused by gbf1 mutation disrupts vascular integrity and causes hemorrhage in zebrafish embryos. J. Biol. Chem. *292*, 2315–2327.

Chia, J., Goh, G., Racine, V., Ng, S., Kumar, P., and Bard, F. (2012). RNAi screening reveals a large signaling network controlling the Golgi apparatus in human cells. Mol. Syst. Biol. *8*, 629.

Citterio, C., Vichi, A., Pacheco-Rodriguez, G., Aponte, A.M., Moss, J., and Vaughan, M. (2008). Unfolded protein response and cell death after depletion of brefeldin A-inhibited guanine nucleotide-exchange protein GBF1. Proc. Natl. Acad. Sci. USA *105*, 2877–2882.

Claude, A., Zhao, B.P., Kuziemsky, C.E., Dahan, S., Berger, S.J., Yan, J.P., Armold, A.D., Sullivan, E.M., and Melançon, P. (1999). GBF1: a novel Golgi-associated BFA-resistant guanine nucleotide exchange factor that displays specificity for ADP-ribosylation factor 5. J. Cell Biol. *146*, 71–84.

Claude, A., Zhao, B.P., and Melançon, P. (2003). Characterization of alternatively spliced and truncated forms of the Arf guanine nucleotide exchange factor GBF1 defines regions important for activity. Biochem. Biophys. Res. Commun. *303*, 160–169.

Dagher, Z., Ruderman, N., Tornheim, K., and Ido, Y. (2001). Acute regulation of fatty acid oxidation and AMP-activated protein kinase in human umbilical vein endothelial cells. Circ. Res. *88*, 1276–1282.

Dekker, R.J., van Soest, S., Fontijn, R.D., Salamanca, S., de Groot, P.G., VanBavel, E., Pannekoek, H., and Horrevoets, A.J.G. (2002). Prolonged fluid shear stress induces a distinct set of endothelial cell genes, most specifically lung Krüppel-like factor (KLF2). Blood *100*, 1689–1698.

Doddaballapur, A., Michalik, K.M., Manavski, Y., Lucas, T., Houtkooper, R.H., You, X., Chen, W., Zeiher, A.M., Potente, M., Dimmeler, S., et al. (2015). Laminar shear stress inhibits endothelial cell metabolism via KLF2-mediated repression of PFKFB3. Arterioscler. Thromb. Vasc. Biol. *35*, 137–145.

Donaldson, J.G., and Jackson, C.L. (2011). Arf Family G proteins and their regulators: roles in membrane transport, development and disease. Nat. Rev. Mol. Cell Biol. *12*, 362–375.

Donaldson, J.G., Cassel, D., Kahn, R.A., and Klausner, R.D. (1992). ADP-ribosylation factor, a small GTP-binding protein, is required for binding of the coatomer protein beta-COP to Golgi membranes. Proc. Natl. Acad. Sci. USA *89*, 6408–6412.

D'Souza-Schorey, C., and Chavrier, P. (2006). ARF proteins: roles in membrane traffic and beyond. Nat. Rev. Mol. Cell Biol. *7*, 347–358.

Farhat, R., Séron, K., Ferlin, J., Fénéant, L., Belouzard, S., Goueslain, L., Jackson, C.L., Dubuisson, J., and Rouillé, Y. (2016). Identification of class II ADP-ribosylation factors as cellular factors required for hepatitis C virus replication. Cell. Microbiol. *18*, 1121–1133.

Farhat, R., Ankavay, M., Lebsir, N., Gouttenoire, J., Jackson, C.L., Wychowski, C., Moradpour, D., Dubuisson, J., Rouillé, Y., and Cocquerel, L. (2018). Identification of GBF1 as a cellular factor required for hepatitis E virus RNA replication. Cell. Microbiol. *20*.

Ferraro, F., Kriston-Vizi, J., Metcalf, D.J., Martin-Martin, B., Freeman, J., Burden, J.J., Westmoreland, D., Dyer, C.E., Knight, A.E., Ketteler, R., et al. (2014). A two-tier Golgi-based control of organelle size underpins the functional plasticity of endothelial cells. Dev. Cell *29*, 292–304.

Ferraro, F., Mafalda Lopes da, S., Grimes, W., Lee, H.K., Ketteler, R., Kriston-Vizi, J., and Cutler, D.F. (2016). Weibel-Palade body size modulates the adhesive activity of its von Willebrand factor cargo in cultured endothelial cells. Sci. Rep. *6*, 32473.

García, I.A., Torres Demichelis, V., Viale, D.L., Di Giusto, P., Ezhova, Y., Polishchuk, R.S., Sampieri, L., Martinez, H., Sztul, E., and Alvarez, C. (2017). CREB3L1-mediated functional and structural adaptation of the secretory pathway in hormone-stimulated thyroid cells. J. Cell Sci. *130*, 4155–4167.

Godi, A., Pertile, P., Meyers, R., Marra, P., Di Tullio, G., Iurisci, C., Luini, A., Corda, D., and De Matteis, M.A. (1999). ARF mediates recruitment of PtdIns-4-OH kinase-beta and stimulates synthesis of PtdIns(4,5)P2 on the Golgi complex. Nat. Cell Biol. *1*, 280–287.

Guo, Y., Walther, T.C., Rao, M., Stuurman, N., Goshima, G., Terayama, K., Wong, J.S., Vale, R.D., Walter, P., and Farese, R.V. (2008). Functional genomic screen reveals genes involved in lipid-droplet formation and utilization. Nature *453*, 657–661.

Gupta, G.D., Swetha, M.G., Kumari, S., Lakshminarayan, R., Dey, G., and Mayor, S. (2009). Analysis of endocytic pathways in Drosophila cells reveals a conserved role for GBF1 in internalization via GEECs. PLoS One *4*, e6768.

Hannah, M.J., Hume, A.N., Arribas, M., Williams, R., Hewlett, L.J., Seabra, M.C., and Cutler, D.F. (2003). Weibel-Palade bodies recruit Rab27 by a content-driven, maturation-dependent mechanism that is independent of cell type. J. Cell Sci. *116*, 3939–3948.

Hannah, M.J., Skehel, P., Erent, M., Knipe, L., Ogden, D., and Carter, T. (2005). Differential kinetics of cell surface loss of von Willebrand factor and its propolypeptide after secretion from Weibel-Palade bodies in living human endothelial cells. J. Biol. Chem. *280*, 22827–22830.

Hansen, M.D., Johnsen, I.B., Stiberg, K.A., Sherstova, T., Wakita, T., Richard, G.M., Kandasamy, R.K., Meurs, E.F., and Anthonsen, M.W. (2017). Hepatitis C virus triggers Golgi fragmentation and autophagy through the immunity-related GTPase M. Proc. Natl. Acad. Sci. USA *114*, E3462–E3471.

Hardie, D.G. (2004). The AMP-activated protein kinase pathway – new players upstream and downstream. J. Cell Sci. *117*, 5479–5487.

Harrison-Lavoie, K.J., Michaux, G., Hewlett, L., Kaur, J., Hannah, M.J., Lui-Roberts, W.W.Y., Norman, K.E., and Cutler, D.F. (2006). P-selectin and CD63 use different mechanisms for delivery to Weibel–Palade bodies. Traffic *7*, 647–662.

Hewlett, L., Zupančič, G., Mashanov, G., Knipe, L., Ogden, D., Hannah, M.J., and Carter, T. (2011). Temperature-dependence of Weibel-Palade body exocytosis and cell surface dispersal of von Willebrand factor and its propolypeptide. PLoS One *6*, e27314.

Huang, R.H., Wang, Y., Roth, R., Yu, X., Purvis, A.R., Heuser, J.E., Egelman, E.H., and Sadler, J.E. (2008). Assembly of Weibel-Palade body-like tubules from N-terminal domains of von Willebrand factor. Proc. Natl. Acad. Sci. USA *105*, 482–487.

Ishii, M., Suda, Y., Kurokawa, K., and Nakano, A. (2016). COPI is essential for Golgi cisternal maturation and dynamics. J. Cell Sci. *129*, 3251–3261.

Kawamoto, K., Yoshida, Y., Tamaki, H., Torii, S., Shinotsuka, C., Yamashina, S., and Nakayama, K. (2002). GBF1, a guanine nucleotide exchange factor for ADP-ribosylation factors, is localized to the cis-Golgi and involved in membrane association of the COPI coat. Traffic *3*, 483–495.

Kuliawat, R., and Arvan, P. (1992). Protein targeting via the "constitutive-like" secretory pathway in isolated pancreatic islets: passive sorting in the immature granule compartment. J. Cell Biol. *118*, 521–529.

Lanke, K.H.W., van der Schaar, H.M., Belov, G.A., Feng, Q., Duijsings, D., Jackson, C.L., Ehrenfeld, E., and van Kuppeveld, F.J.M. (2009). GBF1, a guanine nucleotide exchange factor for Arf, is crucial for coxsackievirus B3 RNA replication. J. Virol *83*, 11940–11949.

Lefrançois, S., and McCormick, P.J. (2007). The Arf GEF GBF1 is required for GGA recruitment to Golgi membranes. Traffic *8*, 1440–1451.

Lopes da Silva, M., and Cutler, D.F. (2016). Von Willebrand factor multimerization and the polarity of secretory pathways in endothelial cells. Blood *128*, 277–285.

Lopes da Silva, M., O'Connor, M.N., Kriston-Vizi, J., White, I.J., Al-Shawi, R., Simons, J.P., Mössinger, J., Haucke, V., and Cutler, D.F. (2016). Type II PI4-kinases control Weibel-Palade body biogenesis and von Willebrand factor structure in human endothelial cells. J. Cell. Sci. *129*, 2096–2105.

Lui-Roberts, W.W.Y., Collinson, L.M., Hewlett, L.J., Michaux, G., and Cutler, D.F. (2005). An AP-1/clathrin coat plays a novel and essential role in forming the Weibel-Palade bodies of endothelial cells. J. Cell Biol. *170*, 627–636.

Maiers, J.L., Kostallari, E., Mushref, M., deAssuncao, T.M., Li, H., Jalan-Sakrikar, N., Huebert, R.C., Cao, S., Malhi, H., and Shah, V.H. (2017). The unfolded protein response mediates fibrogenesis and collagen I secretion through regulating TANGO1 in mice. Hepatology *65*, 983–998.

Malsam, J., Satoh, A., Pelletier, L., and Warren, G. (2005). Golgin tethers define subpopulations of COPI vesicles. Science *307*, 1095–1098.

Manolea, F., Claude, A., Chun, J., Rosas, J., and Melançon, P. (2008). Distinct functions for Arf guanine nucleotide exchange factors at the Golgi complex: GBF1 and BIGs are required for assembly and maintenance of the Golgi stack and trans-Golgi network, respectively. Mol. Biol. Cell *19*, 523–535.

Manolea, F., Chun, J., Chen, D.W., Clarke, I., Summerfeldt, N., Dacks, J.B., and Melançon, P. (2010). Arf3 is activated uniquely at the trans-Golgi network by brefeldin A-inhibited guanine nucleotide exchange factors. Mol. Biol. Cell *21*, 1836–1849.

Mao, L., Li, N., Guo, Y., Xu, X., Gao, L., Xu, Y., Zhou, L., and Liu, W. (2013). AMPK phosphorylates GBF1 for mitotic Golgi disassembly. J. Cell Sci. *126*, 1498–1505.

Mazaki, Y., Nishimura, Y., and Sabe, H. (2012). GBF1 bears a novel phosphatidylinositol-phosphate binding module, BP3K, to link PI3Kγ activity with Arf1 activation involved in GPCR-mediated neutrophil chemotaxis and superoxide production. Mol. Biol. Cell *23*, 2457–2467.

Mihaylova, M.M., and Shaw, R.J. (2011). The AMPK signaling pathway coordinates cell growth, autophagy, and metabolism. Nat. Cell Biol. *13*, 1016–1023.

Miyamoto, T., Oshiro, N., Yoshino, K., Nakashima, A., Eguchi, S., Takahashi, M., Ono, Y., Kikkawa, U., and Yonezawa, K. (2008). AMP-activated protein kinase phosphorylates Golgi-specific brefeldin A Resistance Factor 1 at Thr1337 to induce disassembly of Golgi apparatus. J. Biol. Chem. *283*, 4430–4438.

Monetta, P., Slavin, I., Romero, N., and Alvarez, C. (2007). Rab1b interacts with GBF1 and modulates both ARF1 dynamics and COPI association. Mol. Biol. Cell *18*, 2400–2410.

Morohashi, Y., Balklava, Z., Ball, M., Hughes, H., and Lowe, M. (2010). Phosphorylation and membrane dissociation of the ARF exchange factor GBF1 in mitosis. Biochem. J. *427*, 401–412.

Moss, J., and Vaughan, M. (1995). Structure and function of ARF proteins: activators of cholera toxin and critical components of intracellular vesicular transport processes. J. Biol. Chem. *270*, 12327–12330.

Nightingale, T.D., Pattni, K., Hume, A.N., Seabra, M.C., and Cutler, D.F. (2009). Rab27a and MyRIP regulate the amount and multimeric state of VWF released from endothelial cells. Blood *113*, 5010–5018.

Nogueira, C., Erlmann, P., Villeneuve, J., Santos, A.J., Martínez-Alonso, E., Martínez-Menárguez, J.Á., and Malhotra, V. (2014). SLY1 and syntaxin 18 specify a distinct pathway for procollagen VII export from the endoplasmic reticulum. ELife *3*, e02784.

Novodvorsky, P., and Chico, T.J.A. (2014). The role of the transcription factor KLF2 in vascular development and disease. Prog. Mol. Biol. Transl. Sci. *124*, 155–188.

Papanikou, E., Day, K.J., Austin, J., and Glick, B.S. (2015). COPI selectively drives maturation of the early Golgi. ELife *4*.

Rehemtulla, A., and Kaufman, R.J. (1992). Preferred sequence requirements for cleavage of pro-von Willebrand factor by propeptide-processing enzymes. Blood *79*, 2349–2355.

Sadler, J.E., Budde, U., Eikenboom, J.C.J., Favaloro, E.J., Hill, F.G.H., Holmberg, L., Ingerslev, J., Lee, C.A., Lillicrap, D., Mannucci, P.M., et al. (2006). Update on the pathophysiology and classification of von Willebrand disease: a report of the Subcommittee on von Willebrand factor. J. Thromb. Haemost. *4*, 2103–2114.

Sáenz, J.B., Sun, W.J., Chang, J.W., Li, J., Bursulaya, B., Gray, N.S., and Haslam, D.B. (2009). Golgicide A reveals essential roles for GBF1 in Golgi assembly and function. Nat. Chem. Biol. *5*, 157–165.

Saito, K., Maeda, M., and Katada, T. (2017). Regulation of the Sar1 GTPase cycle is necessary for large cargo secretion from the endoplasmic reticulum. Front Cell Dev. Biol. *5*, 75.

Santos, A.J., Raote, I., Scarpa, M., Brouwers, N., and Malhotra, V. (2015). TANGO1 recruits ERGIC membranes to the endoplasmic reticulum for procollagen export. Elife. *4*, e10982.

Sathanoori, R., Rosi, F., Gu, B.J., Wiley, J.S., Müller, C.E., Olde, B., and Erlinge, D. (2015). Shear stress modulates endothelial KLF2 through activation of P2X4. Purinergic Signal. *11*, 139–153.

Schindelin, J., Arganda-Carreras, I., Frise, E., Kaynig, V., Longair, M., Pietzsch, T., Preibisch, S., Rueden, C., Saalfeld, S., Schmid, B., et al. (2012). Fiji: an open source platform for biological-image analysis. Nat. Methods *9*, 676–682.

Schneider, C.A., Rasband, W.S., and Eliceiri, K.W. (2012). NIH Image to ImageJ: 25 years of image analysis. Nat. Methods *9*, 671–675.

Soni, K.G., Mardones, G.A., Sougrat, R., Smirnova, E., Jackson, C.L., and Bonifacino, J.S. (2009). Coatomer-dependent protein delivery to lipid droplets. J. Cell Sci. *122*, 1834–1841.

Szul, T., and Sztul, E. (2011). COPII and COPI traffic at the ER-Golgi interface. Physiology *26*, 348–364.

Szul, T., Grabski, R., Lyons, S., Morohashi, Y., Shestopal, S., Lowe, M., and Sztul, E. (2007). Dissecting the role of the ARF guanine nucleotide exchange factor GBF1 in Golgi biogenesis and protein trafficking. J. Cell Sci. *120*, 3929–3940.

Volpicelli-Daley, L.A., Li, Y., Zhang, C.J., and Kahn, R.A. (2005). Isoform-selective effects of the depletion of ADP-ribosylation factors 1-5 on membrane traffic. Mol. Biol. Cell *16*, 4495–4508.

Wang, J., Alexanian, A., Ying, R., Kizhakekuttu, T.J., Dharmashankar, K., Vasquez-Vivar, J., Gutterman, D.D., and Widlansky, M.E. (2012). Acute exposure to low glucose rapidly induces endothelial dysfunction and mitochondrial oxidative stress: role for AMP kinase. Arterioscler. Thromb. Vasc. Biol. *32*, 712–720.

Wang, Q., Liang, B., Shirwany, N.A., and Zou, M.H. (2011). 2-Deoxy-D-glucose treatment of endothelial cells induces autophagy by reactive oxygen species-mediated activation of the AMP-activated protein kinase. PLoS One *6*, e17234.

Whitt, M.A., Cox, M.E., Kansal, R., and Cox, J.V. (2015). Kinetically distinct sorting pathways through the Golgi exhibit different requirements for Arf1. Traffic *16*, 267–283.

Wilson, D.G., Phamluong, K., Li, L., Sun, M., Cao, T.C., Liu, P.S., Modrusan, Z., Sandoval, W.N., Rangell, L., Carano, R.A.D., et al. (2011). Global defects in collagen secretion in a Mia3/TANGO1 knockout mouse. J. Cell Biol. *193*, 935–951.

Xi, G., Rosen, C.J., and Clemmons, D.R. (2016). IGF-I and IGFBP-2 stimulate AMPK activation and autophagy, which are required for osteoblast differentiation. Endocrinology *157*, 268–281.

Young, A., Wu, W., Sun, W., Benjamin Larman, H.B., Wang, N., Li, Y.S., Shyy, J.Y., Chien, S., and García-Cardeña, G. (2009). Flow activation of AMP-activated protein kinase in vascular endothelium leads to Krüppel-like factor 2 expression. Arterioscler. Thromb. Vasc. Biol. *29*, 1902–1908.

Zhao, X., Lasell, T.K.R., and Melançon, P. (2002). Localization of large ADP-ribosylation factor-guanine nucleotide exchange factors to different Golgi compartments: evidence for distinct functions in protein traffic. Mol. Biol. Cell *13*, 119–133.

Zhu, Y., Traub, L.M., and Kornfeld, S. (1998). ADP-ribosylation factor 1 transiently activates high-affinity adaptor protein complex AP-1 binding sites on Golgi membranes. Mol. Biol. Cell *9*, 1323–1337.

Zhu, Y., Traub, L.M., and Kornfeld, S. (1999). High-affinity binding of the AP-1 adaptor complex to trans-Golgi network membranes devoid of mannose 6-phosphate receptors. Mol. Biol. Cell *10*, 537–549.

STAR★METHODS

KEY RESOURCES TABLE

REAGENT or RESOURCE	SOURCE	IDENTIFIER
Antibodies		
Rabbit polyclonal anti-VWF propeptide region	Hewlett et al., (2011)	N/A
sheep polyclonal anti-VWF full length protein	Serotec	#AHP062
sheep anti-TGN46	BioRad	#AHP500G
mouse anti-GBF1	BD Biosciences	#612116
rabbit anti-GORASP2	Proteintech	#10598-1-AP
mouse anti-ERGIC53	Enzo	#ENZ-ABS300-0100
rabbit anti-giantin	Abcam	#ab24586
Anti-Clathrin heavy chain	F.M. Brodsky lab	clone X22
mouse anti-p230	BD Biosciences	#611280
mouse anti-GM130	BD Biosciences	#610823
mouse anti-calnexin	Abcam	#ab31290
mouse anti-PDI	Invitrogen	#MA3-018 (RL77
rabbit anti-Calreticulin	Affinity Bioreagents	#PA3-900
sheep anti-P-selectin	R&D Systems	#BBA32
mouse anti-CD63	Abcam	#ab59479
rabbit anti-collagen IV	Abcam	#ab6586
rabbit anti-collagen I	Abcam	#ab34710
rabbit anti-collagen III	Abcam	#ab7778
rabbit anti-collagen VI	Abcam	#ab6588
rabbit anti-fibronectin	Sigma	#F3648
mouse anti-GFP	Roche	#11814460001
rabbit anti-VWF	DAKO	#A0082
mouse monoclonal anti-human VWF	DAKO	#cloneF8
mouse monoclonal anti-β-actin	Santa Cruz	#sc-47778
mouse monoclonal anti-γ-adaptin	BD Biosciences	#610385
mouse monoclonal anti-NP62	BD Biosciences	#610497
rabbit anti-humanGBF1-Thr1337 phosphorylated	Immuno-Biological lab	#28065
Chemicals, Peptides, and Recombinant Proteins		
Brefeldin A (BFA)	Sigma	#B5936
Golgicide A	Sigma	#G0923
nocodazole	Sigma	#M1404
histamine	Enzo	#ALX-550-132-6005
phorbol 12-myristate 13-acetate (PMA)	Sigma	#M1404
Aminoimidazole-4-carboxamide-1-b-d-ribofuranoside (AICAR)	Enzo	#BML-E1330-0050
2 deoxy-D-glucose (2DG)	Sigma	#D6134
D-glucose	Gibco	#A2494001
Experimental Models: Cell Lines		
Human umbilical vein endothelial cells (HUVECs) pooled from donors of both sexes	PromoCell	N/A
Mice stromal fibroblastic reticular cells (FRC)	(Acton et al., 2014)	N/A
Oligonucleotides		
**siRNAs**	N/A	N/A
Dharmacon siGENOME siRNA pools: ARF1	Dharmacon	#M-011580-01

(*Continued on next page*)

Continued

REAGENT or RESOURCE	SOURCE	IDENTIFIER
Dharmacon siGENOME siRNA pools: ARF3	Dharmacon	#M-011581-00
Dharmacon siGENOME siRNA pools: ARF4	Dharmacon	#M-011582-01
Dharmacon siGENOME siRNA pools: ARF5	Dharmacon	#M-011584-01
Dharmacon siGENOME siRNA pools: ARF6	Dharmacon	#M-004008-01
Dharmacon siGENOME siRNA pools: BIG1	Dharmacon	#M-012207-01
Dharmacon siGENOME siRNA pools: BIG2	Dharmacon	#M-012208-02
Dharmacon siGENOME siRNA pools: GBF1	Dharmacon	#M-019783-01
Dharmacon siGENOME siRNA pools: GGA1	Dharmacon	#M-013694-01
Dharmacon siGENOME siRNA pools: GGA2	Dharmacon	#M-012908-01
Dharmacon siGENOME siRNA pools: GGA3	Dharmacon	#M-012881-00
Dharmacon siGENOME siRNA pools: COPA	Dharmacon	#M-011835-01
Dharmacon siGENOME siRNA pools: COPB2	Dharmacon	#M-019847-03
siRNA for VWF (5’-GGGCUCGAGUGUAC CAAAA-3’	Eurofins MWG Operon	(Ferraro et al., 2014)
AP-1 μ1A subunit (5’-AAGGCAUCAAGUAU CGGAAGA-3’)	Eurofins MWG Operon	(Lui-Roberts et al., 2005)
SiRNA Luciferase (“siControl”) (5’-CGUA CGCGGAAUACUUCGA-3’)	Eurofins MWG Operon	(Ferraro et al., 2014)
**rt-PCR primers**	N/A	N/A
QuantiTect Primer assays:	Qiagen	N/A
Hs_ARF1_1_SG	Qiagen	#QT00212688
Hs_ARF3_1_SG	Qiagen	#QT00019887
Hs_ARF4_1_SG	Qiagen	#QT00024731
Hs_ARF5_1_SG	Qiagen	#QT00057939
Hs_ARF6_1_SG	Qiagen	#QT00236824
Hs_ARFGEF1_1_SG (BIG1)	Qiagen	#QT00063581
Hs_ARFGEF2_1_SG (BIG2)	Qiagen	#QT00011620
Hs_GBF1_1_SG	Qiagen	#QT00042399
Hs_GGA1_1_SG	Qiagen	#QT00082159
Hs_GGA2_1_SG	Qiagen	#QT00080122
Hs_GGA3_1_SG	Qiagen	#QT00015162
Hs_COPB2_1_SG	Qiagen	#QT00013097
Hs_CREB3_1_SG	Qiagen	#QT00234472
Hs_SEC13_1_SG	Qiagen	#QT00027657
Recombinant DNA		
Rab27a-GFP	(Nightingale et al., 2009)	N/A
tPa-GFP	(Hewlett et al., 2011)	N/A
Software and Algorithms		
Image J	N/A	https://imagej.nih.gov/ij/
Prism (Graphpad Software)	N/A	https://www.graphpad.com/scientific-software/prism/
Python (v2.7)	N/A	https://www.python.org/download/releases/2.7/
R (i386 3.1.0)	N/A	https://cran.r-project.org/bin/windows/base/old/3.1.0/
ZEN software (2012, version 8.1.6.484, Carl Zeiss, Inc., Germany)	N/A	https://www.zeiss.com/microscopy/int/products/microscope-software/zen-lite.html
iTEM: EMSIS	N/A	https://www.emsis.eu/products/software/item/
Fiji (ImageJ)	N/A	https://fiji.sc/
Amira (version 6.0.0)	N/A	https://www.thermofisher.com/pt/en/home/industrial/electron-microscopy/electron-microscopy-instruments-workflow-solutions/3d-visualization-analysis-software/amira-life-sciences-biomedical.html

(*Continued on next page*)

REAGENT or RESOURCE	SOURCE	IDENTIFIER
Continued		
Photoshop CC (version 2015.5)	N/A	https://www.adobe.com/pt/products/photoshop.html?gclid=EAIaIQobChMImLbhmdGE4QIV1fhRCh06Mg6vEAAYASAAEgKsqfD_BwE&sdid=8DN85NTR&mv=search&ef_id=EAIaIQobChMImLbhmdGE4QIV1fhRCh06Mg6vEAAYASAAEgKsqfD_BwE:G:s&s_kwcid=AL!3085!3!276764797907!b!!g!!%2Bphotoshop%20%2Bcc
MultiQC	N/A	https://multiqc.info/
STAR aligner implemented in BaseSpace (RNA-Seq Alignment pipeline v 1.1.0, Illumina)	N/A	https://www.illumina.com/products/by-type/informatics-products/basespace-sequence-hub/apps/rna-seq-alignment.html
Partek Genomic Suite (v6.6)	N/A	http://www.partek.com/partek-genomics-suite/

CONTACT FOR REAGENT AND RESOURCE SHARING

Further information and requests for resources and reagents should be directed to and will be fulfilled by the Lead Contact, Daniel F. Cutler (d.cutler@ucl.ac.uk).

EXPERIMENTAL MODEL AND SUBJECT DETAILS

Cells

Human umbilical vein endothelial cells (HUVECs) pooled from donors of both sexes were commercially obtained from Lonza or PromoCell. Cells were used within passage 5-6. Cells were maintained in HUVEC Growth Medium (HGM): M199 (Gibco, Life Technologies) supplemented with 20% Fetal Bovine Serum, (Labtech), 30 M g/m L endothelial cell growth supplement from bovine neural tissue (Sigma-Aldrich) and 10 U/m L Heparin (Sigma-Aldrich) and kept at 37°C. Mice stromal fibroblastic reticular cells (FRC) have been described previously (Acton et al., 2014) and were maintained in DMEM plus glucose (Life Technologies, Invitrogen) with 10% FBS, PS and 1% Insulin-Transferrin-Selenium (Life Technologies, Invitrogen).

METHOD DETAILS

Drugs and Drug Treatments

Brefeldin A (BFA) (5 μM) (Sigma #B5936) and Golgicide A (10 μM) (Sigma #G0923) were added to cells in HGM for the times indicated. To unlink the Golgi ribbon cells were incubated in HGM supplemented with nocodazole (1 μg/mL) (Sigma #M1404) for 24 hours. For secretagogue stimulation cells were treated with either histamine (100 μmol/L-100μM) (Enzo #ALX-550-132-6005) or phorbol 12-myristate 13-acetate (PMA) (100 ng/mL) (Sigma #M1404) in Serum Free medium (M199 supplemented with 10 mmol/L HEPES-NaOH, pH 7.4 and 0.1 mg/mL BSA) for 30 mins unless stated. Aminoimidazole-4-carboxamide-1-b-d-ribofuranoside (AICAR) (Enzo #BML-E1330-0050) in HGM was added to cells for 24 hours in the concentration stated. 2 deoxy-D-glucose (2DG) (5.5mM) (Sigma #D6134) was added to cells in HGM for 24 hours. D-glucose (Gibco #A2494001), in the amounts indicated, was added to glucose-free DMEM (Gibco #11966025) for 24 hours.

Microscopy – Antibodies Used

Rabbit polyclonal anti-VWF pro-peptide region which only stains processed VWF (termed 'pro-VWF' throughout) was produced as in (Hewlett et al., 2011), sheep polyclonal anti-VWF full length protein which stains both processed and unprocessed-VWF (termed 'unp-VWF' throughout) (Serotec #AHP062), sheep anti-TGN46 (BioRad #AHP500G), mouse anti-GBF1 (BD Biosciences #612116), rabbit anti-GORASP2 (Proteintech #10598-1-AP), mouse anti-ERGIC53 (Enzo #ENZ-ABS300-0100), rabbit anti-giantin (Abcam #ab24586), anti-Clathrin heavy chain (clone X22, kind gift from F.M. Brodsky), mouse anti-p230 (BD Biosciences #611280), mouse anti-GM130 (BD Biosciences #610823), mouse anti-calnexin (Abcam #ab31290), mouse anti-PDI (Invitrogen #MA3-018 (RL77)), rabbit anti-Calreticulin (Affinity Bioreagents #PA3-900), sheep anti-P-selectin (R&D Systems #BBA32), mouse anti-CD63 (Abcam #ab59479), rabbit anti-collagen IV (Abcam #ab6586), rabbit anti-collagen I (Abcam #ab34710), rabbit anti-collagen III (Abcam #ab7778), rabbit anti-collagen VI (Abcam #ab6588), rabbit anti-fibronectin (Sigma #F3648), mouse anti-GFP (Roche #11814460001). Alexa-Fluor secondary antibodies were from Invitrogen. All microscopy experiments were repeated at least 3 times.

To visualise Rab27a and tPa, cells were nucleofected with Rab27a-GFP (Nightingale et al., 2009) or tPa-GFP (Hewlett et al., 2011) and allowed to express for 20-24 hours. Cells were fixed and stained with anti-GFP antibody and only low expressing cells were imaged to avoid overexpression artefacts. DNAs were delivered to cells using an AMAXA Nucleofector II (Lonza). Constant number of HUVECs was used per reaction (1 millions) and between 1-5 mg of DNA was used per reaction.

Microscopy - Confocal

Cells were grown on gelatin-coated coverslips or 96-well plates then fixed with 4% formaldehyde in PBS for 10 min at RT. Cells were permeabilised (0.2% TX-100 in PBS for 10 min) and blocked in 1% bovine serum albumin (BSA) in PBS. Samples were incubated with primary followed by secondary antibodies in 1% BSA. Coverslips were mounted in ProLong Gold antifade reagent with DAPI (Life Technologies) while samples in 96-well plates were kept in PBS and nucleus visualise with, Hoechst 33342 (Life Technologies, H3570). Coverslips were imaged with a 63x (NA 1.3) oil immersion objective in a Leica SP5 confocal system (Leica), and images were analysed using ImageJ(Schneider et al., 2012). 96-well plate images were imaged using an Opera High Content Screening System (Perkin Elmer) using a 40x air objective (NA 0.6). For each condition, 4-8 replicate wells with 9 fields of view per well were acquired (around 300 cells per well). To quantify the number of VWF exit sites, cells were incubated with rabbit anti-VWF (DAKO #A0082) in the presence of histamine for 15 minutes prior to fixation and immunofluorescence staining (without permeabilisation) with secondary fluorescent antibodies and stained with Hoechst. Unless otherwise stated, confocal images shown are maximum intensity projections of a stack of images.

Microscopy – Super-resolution Structured Illumination Microscopy (SR-SIM)

SR-SIM imaging was performed using Plan-Apochromat 63x/1.4 oil DIC M27 objective, in an Elyra PS.1 microscope (Zeiss). Images were acquired using 5 phase shifts and 3 grid rotations, with the 647 nm, the 561 nm, the 488 nm lasers. A pco.egde sCMOS camera (PCO) was used for acquisition and the resulting images were processed using the ZEN software (2012, version 8.1.6.484, Carl Zeiss, Inc., Germany). For channel alignment, a multicolored bead slide was imaged using the same image acquisition settings and used for the alignment of the different channels. Images shown in Figure are maximum intensity projections of image stacks (unless stated).

Microscopy - High-Throughput Morphometry (HTM) Analysis

Image processing, organelle segmentation and measurement of morphological parameters have been described in detail elsewhere (Ferraro et al., 2014). Briefly, images of the various channels (i.e., WPBs, Nucleus, VWF exit sites) were segmented using Python (v2.7) (code available upon request). Data analysis was done using R (i386 3.1.0). Mean number of WPBs per cell was calculated by counting the number of VWF-positive objects and dividing by the number of nuclei for each field of view. Mean number of VWF exit-sites per cell was calculated by counting the number of VWF-positive objects and divide by the number of nuclei for each field of view. Mean number of VWF 'quanta' per cell was calculated by summing all WPB ferets in each image, dividing by 0.5 (mean size of each VWF quanta in μm), and divide by the number of nuclei for each field of view. To represent changes in WPB size, the longest axis (feret diameter) of each VWF-positive object was measured and the percentage of the number of objects whose feret diameter is longer than 2 μm was calculated from the entire population for each condition (usually more than 100,000 objects per condition per experiment). To infer the amount of unprocessed VWF by image analysis, cells were stained for VWF (staining only WPBs) and full length VWF (showing unprocessed and VWF inside WPBs). The VWF channel was subtracted from the full length VWF channel, removing all WPB objects from the resulting image. The mean fluorescence intensity was calculated and divided by the number of nuclei in each field of view. The number of VWF exit sites per cell was calculated by segmenting VWF exocytic structures and dividing by the number of nuclei for each field of view.

To quantify Golgi area cells were stained with anti-TGN46 antibody and the same segmentation was used with appropriate setting to segment the Golgi.

Microscopy – Correlative Light and Electron Microscopy (CLEM)

HUVECs were transfected with GFP-VWF (Ferraro et al., 2014) and seeded on gelatine-coated photoetched gridded coverslips (MatTek Corporation). After 24 hours, cells were fixed with 2% PFA (TAAB Laboratory Equipment Ltd) with 0.1% Gluteraldehyde (TAAB Laboratory Equipment Ltd) in PBS for 30 mins before image acquisition using an inverted Leica SP5 (63x lens, NA 1.3, oil immersion). Samples were subsequently washed with 0.1M Sodium Cacodylate, osmicated and further processed for resin embedding (Lopes da Silva and Cutler, 2016). Resin blocks were sectioned (Leica Microsystems) and 70-nm ultrathin serial sections collected on formvar-coated slot grids were stained with lead citrate and observed with a transmission electron microscope, Tecnai G2 Spirit (FEI) equipped with a Morada CCD camera (Olympus-SIS). Serial electron microscope (EM) image alignment was performed using the TrakEM2 plugin (Cardona et al., 2012) in Fiji (Schindelin et al., 2012), with manual modification using Amira (version 6.0.0) if required. EM and light microscopy data sets were registered manually in Photoshop CC (version 2015.5) using DIC images and serial EM images, where nuclear and plasma membrane features, together with lipid droplets were used as unbiased fiducials. EM image segmentation, 3D reconstruction and rendering was performed using Amira (version 6.0.0). Image panels were assembled using Fiji and Photoshop CC (version 2015.5).

siRNA Treatment

siRNAs were delivered to cells using an AMAXA Nucleofector II (Lonza). Constant numbers of HUVECs were used per reaction (1 millions in 1st round and 1.5 million in 2nd round). Cells were typically nucleofected twice (two days apart) for optimal gene ablation and were processed 48 hours after the second round. The following Dharmacon siGENOME siRNA pools were used (amount per reaction):

ARF1 (200 pmol #M-011580-01), ARF3 (200 pmol #M-011581-00), ARF4 (100 pmol #M-011582-01), ARF5 (300 pmol #M-011584-01), ARF6 (200 pmol #M-004008-01), BIG1 (100 pmol #M-012207-01), BIG2 (200 pmol #M-012208-02), GBF1 (200 pmol #M-019783-01), GGA1 (400 pmol #M-013694-01), GGA2 (400 pmol #M-012908-01), GGA3 (400 pmol #M-012881-00), COPI-α ('COPA', 100 pmol #M-011835-01), COPI-β' ('COPB2', 100 pmol #M-019847-03).Custom oligo were synthesised (Eurofins MWG Operon) for VWF (50 pmol, 5'-GGGCUCGAGUGUACCAAAA-3') and AP-1 μ1A subunit (300 pmol, 5'-AAGGCAUCAAGUAUCG GAAGA-3'). For each experiment, control cells were either mock nucleofected in siRNA buffer or nucleofected with siRNA targeting Luciferase (100 pmol, 5'-CGUACGCGGAAUACUUCGA-3').

Quantitative PCR

RNA was prepared from treated HUVECs using an RNeasy kit (Qiagen), and equal amounts of RNA were used to prepare cDNA using the SuperScript III first-strand synthesis system (Life technologies). The following primers were used: VWF forward 5′-GCCAT CATGCATGAGGTCAGA-3′and reverse 5′-GGCTCCGTTCTCATCACAGAT-3′, actin forward 5′-TGGTGGTGAAGCTGTAGCC-3′ and reverse 5′-GCGAGAAGATGACCCAGAT-3′. The following QuantiTect Primer assays (Qiagen) were used: Hs_ARFGEF1_1_SG (BIG1) (#QT00063581),Hs_ARFGEF2_1_SG (BIG2) (#QT00011620),Hs_ARF1_1_SG (#QT00212688),Hs_ARF3_1_SG (#QT00019887), Hs_ARF4_1_SG (#QT00024731), Hs_ARF5_1_SG (#QT00057939), Hs_ARF6_1_SG (#QT00236824), Hs_COPB2_1_SG (#QT00013097), Hs_CREB3_1_SG (#QT00234472), Hs_GBF1_1_SG (#QT00042399), Hs_GGA1_1_SG (#QT00082159), Hs_GGA2_1_SG (#QT00080122), Hs_GGA3_1_SG (#QT00015162), Hs_SEC13_1_SG (#QT00027657). DNA amplification was monitored by incorporation of SYBR green (DyNAmo SYBR Green qPCR Kit, Thermo Scientific) and analysed on a Mastercycler ep Realplex thermocycler (Eppendorf). Gene expression was assessed using the $\Delta\Delta$CT method and normalized to actin.

VWF Secretion Assay

WPB secretion was stimulated by challenging HUVECs with histamine or PMA in serum free medium (SFM) for 30 min. Lysates were collect in SFM supplemented with 0.2% protease inhibitors (Sigma-Aldrich) and 0.5% Triton X100. VWF content in cell lysates and releasates was measured using a sandwich ELISA as described previously (Ferraro et al., 2016). Amount of VWF secreted during stimulation is represented as a percentage of total VWF in cells. To pool multiple independent experiments results are shown as a percent of VWF release when compared to the control sample in each experiment.

To measure the amount of constitutively secreted VWF, cells were washed and treated with control or Brefeldin A (BFA) (5 μM) (Sigma #B5936) for 1 hour to block constitutive secretion. Cells were washed again and left to secrete for 1 hour (with and without BFA). The supernatant was collected and the amount of VWF was measured as above. The amount of constitutive VWF secreted is the amount that was blocked when compared with non-BFA treated cells.

lumGFP Secretion and GFP ELISA

lumGFP plasmid DNA has been described previously (Blum et al., 2000). Cells were nucleofected with 20mg of plasmid per reaction (around 1x10^6 cells per reaction). For lumGFP experiments, cells were analysed 24h after nucleofection.

lumGFP secretion collection was performed by washing and incubating confluent cells in SF for 60 minutes. Cells were then lysed to determine total lumGFP levels. Relative amounts of GFP were determined by sandwich ELISA using MaxiSorp plates (Thermo Fisher Scientific) coated with sheep anti-GFP (BioRad), followed by blocking then incubation with samples. Plates were washed and incubated with rabbit anti-GFP (Invitogen) followed by washing and a final incubation with goat anti-Rabbit conjugated with horseradish peroxidase (HRP) (Jackson Laboratories). Plates were developed with o-phenylenediamine dihydrochloride and hydrogen peroxide in a citrate phosphate buffer. Absorbance was analysed at 450 nm in a Thermomax microplate reader (Molecular Devices) using a kinetic protocol with a reading every 30 s for 30 min. A standard curve was made using a lumGFP nucleofected lysates serially diluted. Results are shown as % of secreted GFP from the total GFP measured in each sample (secreted plus lysate).

Western Blotting / Immunoprecipitation

Whole cell lysates were prepared using SDS-lysis buffer (0.5% SDS, 2 mmol/L EDTA, 50 mmol/L NaF, 50 mmol/L Tris/HCl pH 8.0) supplemented with 0.2% protease inhibitors (Sigma-Aldrich). Lysates' DNA was sheared by passing through a syringe needle several times. Protein concentration in lysates was measured with the Pierce BCA Protein Assay Kit (Thermo Scientific). Equal amounts of lysates, by protein, were denatured and reduced in Laemmli buffer, fractionated by SDS-PAGE and electro-blotted on PVDF membranes (Millipore). After blocking with BSA/PBS-T (5% BSA, 0.05% Tween-20 in PBS), membranes were incubated with antibodies diluted in BSA/PBS-T, followed by the appropriate HRP-conjugated secondary antibodies (Jackson Immunoresearch Laboratories). Chemoluminescent signals generated using the Luminata™ Crescendo Western HRP-substrate (Millipore) were digitally acquired with an ImageQuant LAS 4000 CCD system (GE Healthcare Life Sciences) and quantified with ImageJ.

For immunoprecipitations confluent monolayers of HUVECs grown on 10cm dishes were incubated with media containing either 0.1mM or 5mM glucose and with or without AICAR (1mM) for 24 hours. Samples were lysed in 600μl RIPA buffer (150mM NaCl, 1% NP40, 0.5% sodium deoxycholate, 0.1% SDS, 50mM Tris pH 8.0 with, 2mM imidazole, 1mM sodium fluoride, 1.15mM sodium molybdate, 1mM sodium orthovanadate, 1mM HALT phosphatase inhibitor (Thermo) and protease inhibitor cocktail (Sigma)). Samples were centrifuged at 15000rpm for 15 minutes and supernatant incubated with 1μl GBF1 antibody (BD Biosciences) rotating at 4°C for 1 hour 30 minutes followed by addition of 100μl of a 1:1 slurry of protein G sepharose (abcam) for a further 1 hour 30 minutes.

Samples were washed in RIPA buffer three times and in PBS twice. Samples were resuspended in 50μl 2x reducing SDS buffer and incubated at 95°C for 5 minutes to elute protein. Blotting was carried out as described above using 7.5% gels.

Antibodies used for western blotting: mouse anti-human GBF1 (BD Biosciences #612116); rabbit anti-humanGBF1-Thr1337 phosphorylated (Immuno-Biological lab. #28065); mouse monoclonal anti-human VWF, raised to the mature portion of human VWF (Dako #cloneF8); mouse monoclonal anti-β-actin (Santa Cruz #sc-47778); mouse monoclonal anti-γ-adaptin (BD Biosciences #610385); mouse monoclonal anti-NP62 (BD Biosciences #610497).

RNAseq

HUVECS were treated with 200 pmol of either siRNA targeting Luciferase or GBF1 (2 rounds of nucleofection). RNA from cells was collected using an RNeasy kit (Qiagen). RNA was quantified using a Nanodrop (Thermofisher Scientific) and quality analysis was performed using a Bioanalysar (Agilent). 100 ng of total RNA was used as a template for NEB Ultra directional RNA library prep kit with mRNA selection (New England Biolabs). Library quantification (QuBit, Thermofisher Scientific) and quality assessment (Tape Station, D1000 tape, Agilent) were performed. Sequencing was performed using a NextSeq 500 System (Illumina) with High-Output run and 75 bp paired end. Sequencing quality control was done using MultiQC (http://multiqc.info/). Sequence Fastq files were aligned to the reference genome HG19 using STAR aligner implemented in BaseSpace (RNA-Seq Alignment pipeline v 1.1.0, Illumina). BAM file outputs from STAR were annotated using Partek Genomic Suite (v6.6) and the RefSeq data base (RefSeq 21). Differential analysis (control vs siGBF1 samples) was performed using Partek Genomic Suite (v6.6) running ANOVA (n=4 independent replicates). Only hits with a threshold criteria for significance of p-value <0.05 and fold change of >2 were considered. Entire list of hits and individual p-values is shown in Table S1.

Pathway Enrichment Analysis

The 115 RNAseq genes which passed the criteria (of p-value <0.05 and fold change of >2) were analysed using Gene Ontology Consortium PANTHER Version 12.0 (released 2017-10-24). (http://www.geneontology.org/page/go-enrichment-analysis). PANTHER overrepresentation Test (release 20170413) was used and our gene list was compared with Homo Sapiens GO Ontology database (released 2017-08-14). The only overrepresented biological processes were a 15.78-fold increase (p-value 3.32E-03, with Bonferroni correction for multiple testing) in retrograde vesicle-mediated transport (Golgi-to-ER) components.

QUANTIFICATION AND STATISTICAL ANALYSIS

Data are expressed as means +S.E.M, unless otherwise stated. Statistically significant differences between different groups were determined using unpaired two-tailed *t*-test or one-way ANOVA, with Dunnett's multiple comparisons test or a two-way ANOVA with a Sidak's multiple comparisons test. P values < 0.05 were considered statistically significant. Significances are represented in the Figures as follows: n.s, $P > 0.05$; *$P < 0.05$; **$P < 0.01$; ***$P < 0.001$; ****$P < 0.0001$, unless individual P values as stated. All statistical tests were carried out in GraphPad Prism (version 6), except for the two-sample Kolmogorov-Smirnov test performed on the Cumulative frequency curve of Golgi area in Figure S2D which was performed in RStudio (Version 1.1.463).

Developmental Cell, Volume *49*

Supplemental Information

A GBF1-Dependent Mechanism for Environmentally Responsive Regulation of ER-Golgi Transport

Mafalda Lopes-da-Silva, Jessica J. McCormack, Jemima J. Burden, Kimberly J. Harrison-Lavoie, Francesco Ferraro, and Daniel F. Cutler

Figure S1

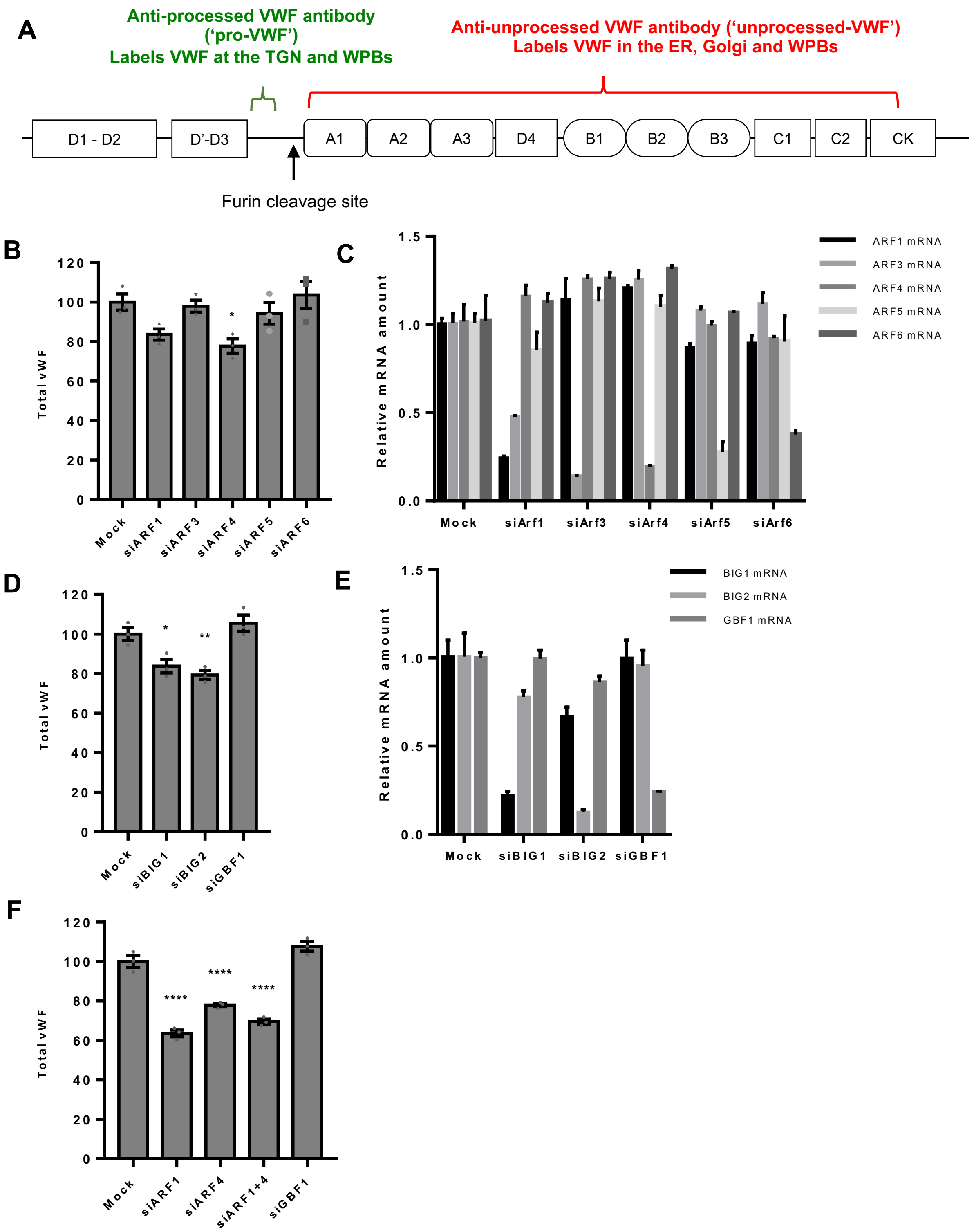
A
Anti-processed VWF antibody
('pro-VWF')
Labels VWF at the TGN and WPBs
Anti-unprocessed VWF antibody ('unprocessed-VWF')
Labels VWF in the ER, Golgi and WPBs
D1 - D2
D'-D3
A1
A2
A3
D4
B1
B2
B3
C1
C2
CK
Furin cleavage site
B
Total vWF
Mock
siARF1
siARF3
siARF4
siARF5
siARF6
C
Relative mRNA amount
ARF1 mRNA
ARF3 mRNA
ARF4 mRNA
ARF5 mRNA
ARF6 mRNA
Mock
siArf1
siArf3
siArf4
siArf5
siArf6
D
Total vWF
Mock
siBIG1
siBIG2
siGBF1
E
Relative mRNA amount
BIG1 mRNA
BIG2 mRNA
GBF1 mRNA
Mock
siBIG1
siBIG2
siGBF1
F
Total vWF
Mock
siARF1
siARF4
siARF1+4
siGBF1

Figure S1

Figure S1. *Related to Figure 1.*

A. Diagram showing the various domains of VWF protein and antigen sites for the two different VWF antibodies used throughout this study.

B. Total amount of VWF protein in siRNA targeting ARF proteins treated cells, relative to control, measured by ELISA. N=3 independent experiments, S.E.M., one-way ANOVA with Dunnett's multiple comparisons test, *=p=0.0186.

C. rtPCR showing knockdown down of mRNA upon treatment with various siRNAs.

D. Total amount of VWF protein in siRNA targeting ARF GEF proteins treated cells, relative to control, measured by ELISA. N=3 independent experiments, S.E.M., one-way ANOVA with Dunnett's multiple comparisons test, *=p=0.0218, **=p=0.0058.

E. rtPCR showing knockdown down of mRNA upon treatment with various siRNAs.

F. Total amount of VWF protein in siRNA treated cells, relative to control, measured by ELISA. N=3 independent experiments, S.E.M., one-way ANOVA with Dunnett's multiple comparisons test, ****=P<0.0001.

Figure S2

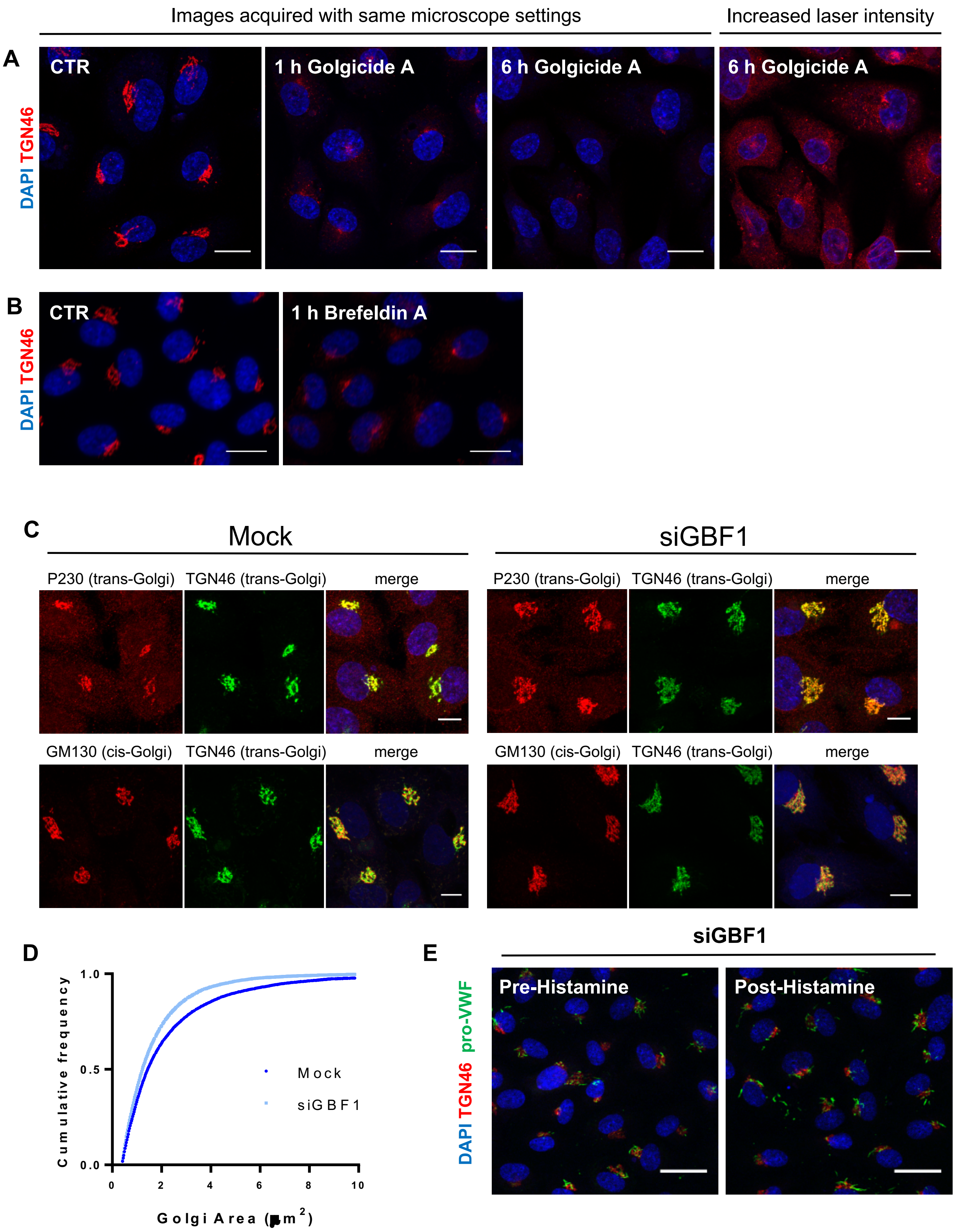

Images acquired with same microscope settings
Increased laser intensity
A
DAPI TGN46
CTR
1 h Golgicide A
6 h Golgicide A
6 h Golgicide A
B
DAPI TGN46
CTR
1 h Brefeldin A
C
Mock
siGBF1
P230 (trans-Golgi)
TGN46 (trans-Golgi)
merge
GM130 (cis-Golgi)
TGN46 (trans-Golgi)
merge
D
Cumulative frequency
1.0
0.5
0.0
0
2
4
6
8
10
Golgi Area (µm²)
Mock
siGBF1
E
siGBF1
DAPI TGN46 pro-VWF
Pre-Histamine
Post-Histamine

Figure S2

Figure S2. ***Related to Figure 1 and Figure 6.***

A. Immunofluorescence confocal images of HUVECs treated with Golgicide A for different lengths of time (hours) showing TGN46 (red) and DAPI (blue). Scale bars: 20 μm.

B. Immunofluorescence images of HUVECs treated with Brefeldin A for 1 hour showing TGN46 (red) and DAPI (blue). Scale bars: 20 μm.

C. Immunofluorescence images of control and GBF1 siRNA treated HUVECs for various ER-Golgi proteins. Scale bars: 10 μm.

D. Cumulative frequency graph showing the increase in area occupied by the Golgi in GBF1-ablated cells when compared with Mock cells. Two-sample Kolmogorov-Smirnov test, ****=P= 2.2e-16.

E. Immunofluorescence images of GBF1-ablated cells, pre and post-Histamine stimulation (30 mins), showing that most WPBs remain inside the cells, in the peri-Golgi area, and are not secreted by cells. Processed-VWF (green), TGN46 (red) and DAPI (blue). Scale bars: 30 μm.

Figure S3

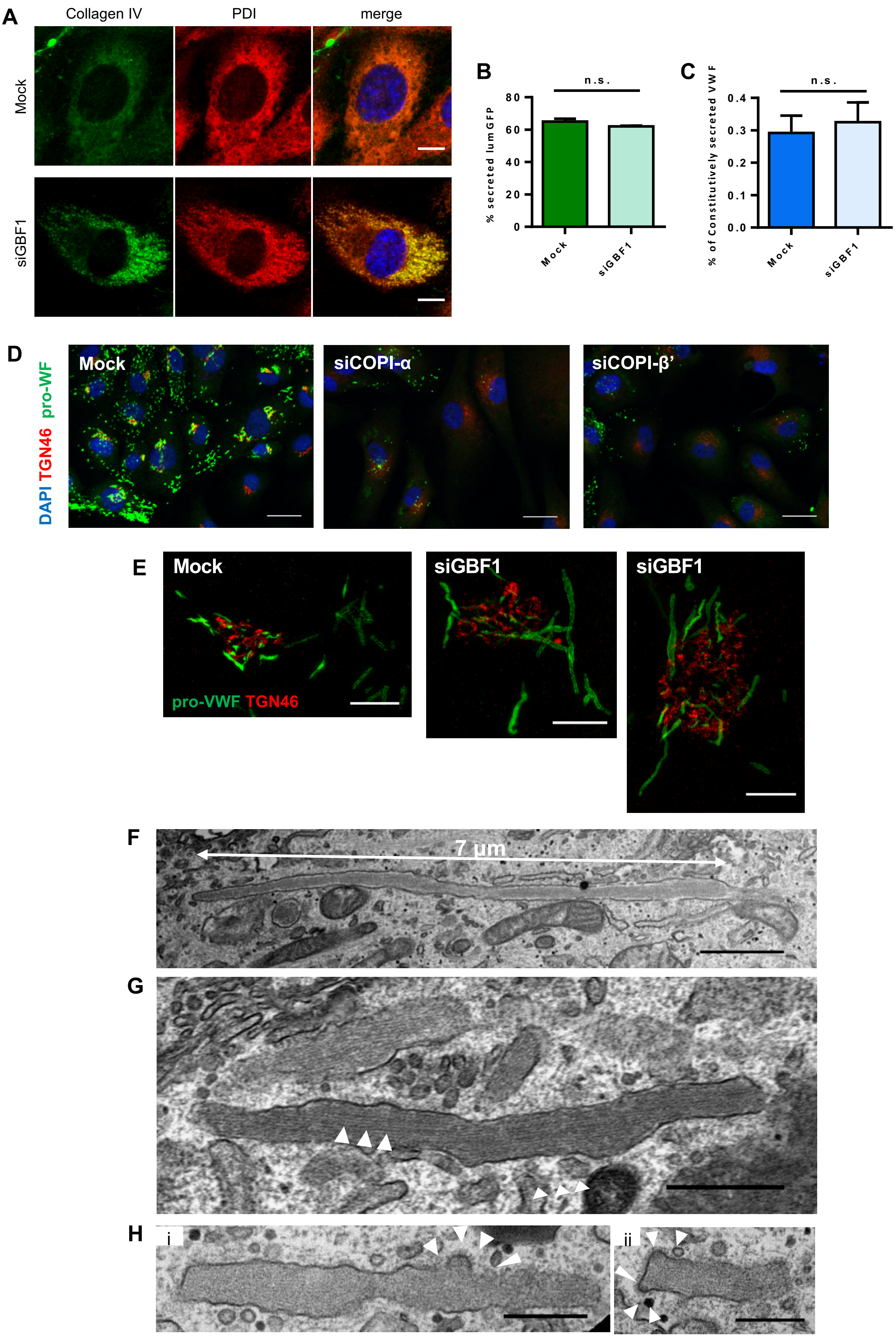
A
Collagen IV
PDI
merge
Mock
siGBF1
B
n.s.
% secreted lumGFP
80
60
40
20
0
Mock
siGBF1
C
n.s.
% of Constitutively secreted VWF
0.4
0.3
0.2
0.1
0.0
Mock
siGBF1
D
DAPI TGN46 pro-WF
Mock
siCOPI-α
siCOPI-β’
E
Mock
siGBF1
siGBF1
pro-VWF TGN46
F
7 µm
G
H
i
ii

Figure S3

Figure S3. Related to Figure 2 and Figure 5.

A. Immunofluorescence images of control and GBF1-ablated HUVECs stained with collagen IV (green) and the ER luminal marker PDI (red) and DAPI (blue). Scale bars: 20 μm.

B. Control and GBF1-ablated cells were transfected with lumGFP construct and the amount of constitutively secreted GFP was measured. N=3 independent experiments, S.E.M., T-test, n.s.= not significant.

C. Proportion of constitutively secreted VWF from total VWF in control and GBF1 siRNA treated cells. N=3 independent experiments, S.E.M., T-test, n.s.= not significant.

D. Immunofluorescence images of control, COPI-α and COPI-β' siRNA treated HUVECs showing pro-VWF (green), TGN46 (red) and DAPI (blue). Scale bars: 30 μm.

E. Super-Resolution Structured Illumination Microscopy (SR-SIM) reconstruction of control and GBF1 siRNA treated cells stained for TGN46 (red) and processed-VWF (green) and DAPI (blue), showing cells with VWF-positive structures of varying lengths. Scale bars: 5 μm.

F. Transmission EM image of a GBF1 siRNA treated cell showing an extremely long WPB, commonly found in GBF1-ablated cells but very rarely in control cells. Scale bar: 1 μm.

G. Transmission EM image of a GBF1 siRNA treated cell showing a WPB where the VWF striations can be observed (arrows). Scale bar: 500 nm.

H. Transmission EM serial sections of a GBF1 siRNA treated cell showing area where clathrin coats are visible (arrows), suggesting that clathrin is still recruited to these WPBs. Scale bar: (i) 300 nm, (ii) 300 nm.

Figure S4

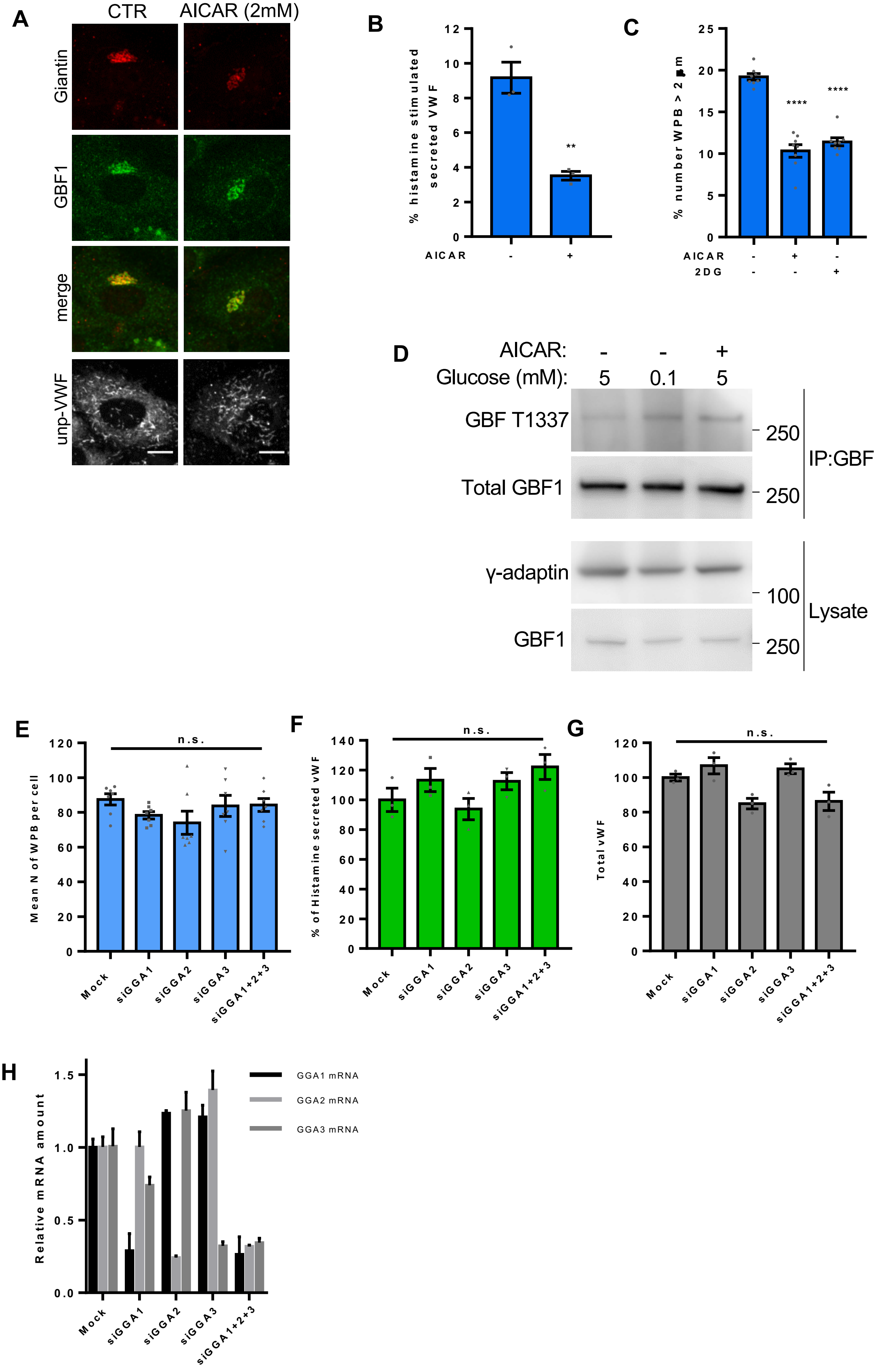

A
CTR
AICAR (2mM)
Giantin
GBF1
merge
unp-VWF
B
% histamine stimulated secreted VWF
AICAR
-
+
**
C
% number WPB > 2 µm
AICAR
2DG
****
****
D
AICAR:
Glucose (mM):
5
0.1
5
GBF T1337
Total GBF1
γ-adaptin
GBF1
250
250
100
250
IP:GBF
Lysate
E
Mean N of WPB per cell
n.s.
Mock
siGGA1
siGGA2
siGGA3
siGGA1+2+3
F
% of Histamine secreted vWF
n.s.
G
Total vWF
n.s.
H
Relative mRNA amount
GGA1 mRNA
GGA2 mRNA
GGA3 mRNA

Figure S4

Figure S4. Related to Figure 7.

A. Immunofluorescence images of control and AICAR (2 mM) treated cells stained with giantin (red), GBF1 (green) and unprocessed-VWF (grey) antibodies. Scale bar: 0 μm.

B. HUVECS were treated with 2 mM AICAR for 24 hours prior to VWF secretion assay. Proportion of secreted VWF from total VWF in control and AICAR treated cells, upon 30 minutes histamine stimulation. N=3 independent experiments, S.E.M., unpaired T-test, **=p=0.0037.

C. HUVECs were treated with 2 mM AICAR or 5.5 mM 2-deoxuglucose (2DG) for 24 hours prior to fixation. The length of WPB was measured by HTM analysis and the graph shows the proportion of WPB in each population with a long axis longer than 2 μm. Both treatments induce the production of shorter WPBs. N=8, one-way ANOVA with Dunnett's multiple comparisons test, ****=p<0.0001.

D. Immunoprecipitation of cells treated with either control (5 mM glucose), low glucose (0.1 mM) or AICAR (2 mM).

E. Mean number of WPB per cells in siRNA targeting GGA proteins treated cells. N=7 wells where for each well the mean for each of 9 fields of view were analysed, S.E.M., one-way ANOVA with Dunnett's multiple comparisons test, n.s.= not significant.

F. Proportion of secreted VWF from total VWF in control and siRNA treated cells, upon 30 minutes histamine stimulation. Results standardised to amount secreted by control cells. N=3 independent experiments, S.E.M., one-way ANOVA with Dunnett's multiple comparisons test, n.s.= not significant.

G. Total amount of VWF protein in siRNA treated cells, relative to control, measured by ELISA. N=3 independent experiments, one-way ANOVA with Dunnett's multiple comparisons test, n.s.= not significant.

H. rtPCR showing knockdown down of mRNA upon treatment with various siRNAs.

Figure S5

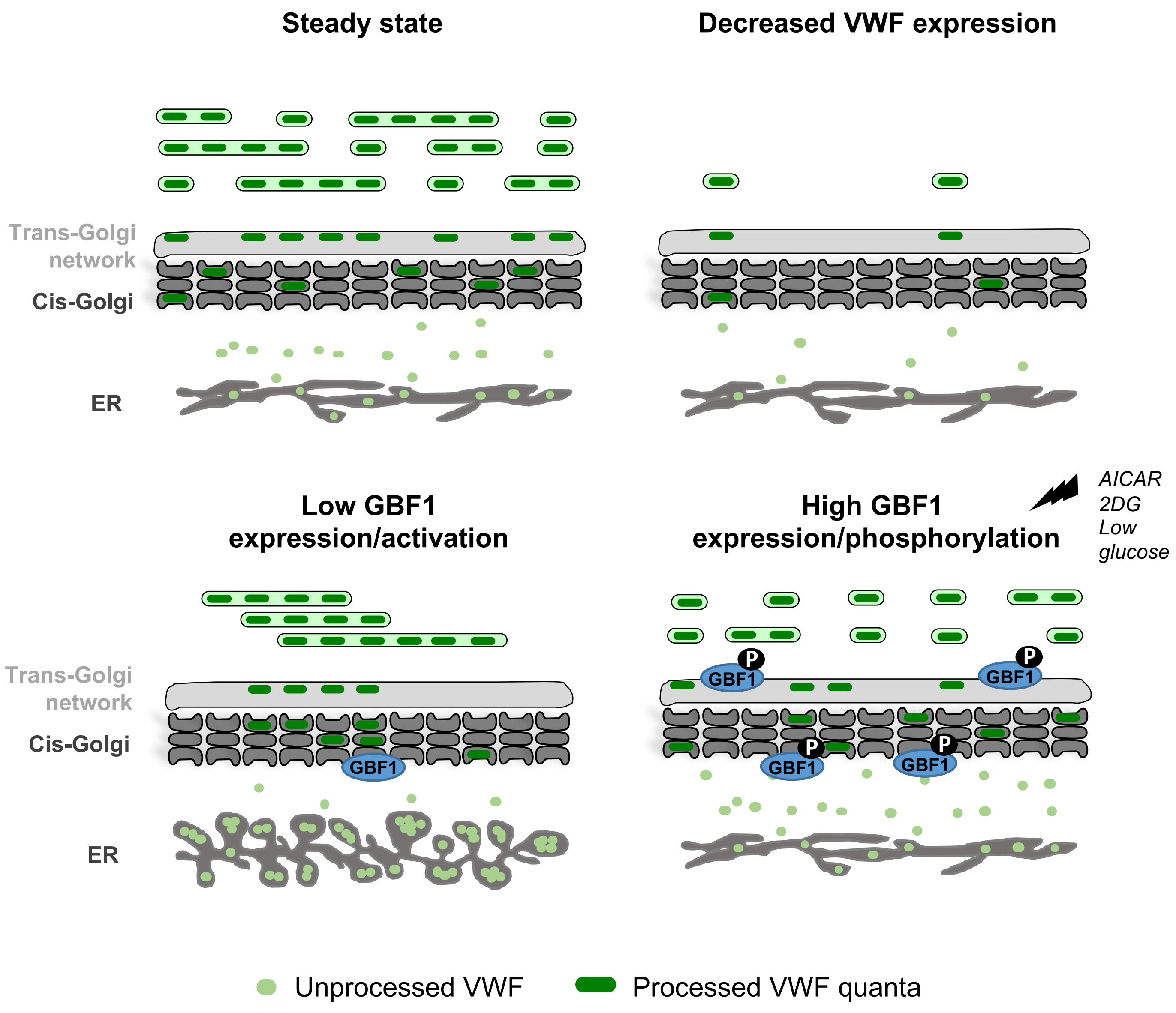

Steady state
Decreased VWF expression
Trans-Golgi network
Cis-Golgi
ER
Low GBF1 expression/activation
High GBF1 expression/phosphorylation
AICAR
2DG
Low glucose
GBF1
P
Unprocessed VWF
Processed VWF quanta

Figure S5

Figure S5. Related to Figures 1-7.

Model of how different sized WPBs are formed under different experimental conditions. Under steady-state conditions, VWF quanta (dark green rods) travel through the cis-Golgi (dark grey) to the continuous lumen of the TGN (light grey) and are packaged into WPBs, the size of which is determined by the number of packaged quanta. If VWF expression is lowered, then the probability of adjacent VWF quanta reaching the TGN at the same time is decreased, and more small WPBs are made. Under low GBF1 expression/activation, the number of recruited COPI-vesicles is decreased, resulting in a reduced rate of Golgi membrane retrieval and hence the rate of Golgi maturation and anterograde trafficking is also reduced. This results in a VWF exit from the ER being delayed, leading to an accumulation of unprocessed VWF (light green circles) in the ER. Since TGN progression and exit is also reduced, this leads to the accumulation of multiple VWF quanta at the TGN therefore increasing the probability of co-packaging into the same, extremely long WPB. When GBF1 is activated, via AMPK signaling (via AICAR, 2DG or glucose starvation), anterograde traffic is increased resulting in increased VWF trafficking through the ER-Golgi and the formation of smaller WPBs.
